# Adaptive selection in the evolution of programmed cell death-1 and its ligands in vertebrates

**DOI:** 10.18632/aging.102827

**Published:** 2020-02-11

**Authors:** Hafiz Ishfaq Ahmad, Jiabin Zhou, Muhammad Jamil Ahmad, Gulnaz Afzal, Haiying Jiang, Xiujuan Zhang, Abdelmotaleb A. Elokil, Musarrat Abbas Khan, Linmiao Li, Huiming Li, Liu Ping, Jinping Chen

**Affiliations:** 1 Guangdong Key Laboratory of Animal Conservation and Resource Utilization, Guangdong Public Laboratory of Wild Animal Conservation and Utilization, Guangdong Institute of Applied Biological Resources, Guangzhou, Guangdong, China; 2 College of Animal Science and Technology, Huazhong Agricultural University, Wuhan, China; 3 Department of Zoology, The Islamia University, Bahawalpur, Pakistan; 4 Animal Production Department, Faculty of Agriculture, Benha University, Moshtohor, Egypt; 5 Department of Animal Breeding and Genetics, Cholistan University of Veterinary and Animal Sciences, Bahawalpur, Pakistan

**Keywords:** PD1, PD-L1, adaptive selection, evolution, vertebrates

## Abstract

Programmed cell death-1 (PD-1) and its ligands, particularly PD-L1 and PD-L2, are the most important proteins responsible for signaling T-cell inhibition and arbitrating immune homeostasis and tolerance mechanisms. However, the adaptive evolution of these genes is poorly understood. In this study, we aligned protein-coding genes from vertebrate species to evaluate positive selection constraints and evolution in the PD1, PD-L1 and PD-L2 genes conserved across up to 166 vertebrate species, with an average of 55 species per gene. We determined that although the positive selection was obvious, an average of 5.3% of codons underwent positive selection in the three genes across vertebrate lineages, and increased positive selection pressure was detected in both the Ig-like domains and transmembrane domains of the proteins. Moreover, the PD1, PD-L1 and PD-L2 genes were highly expressed in almost all tissues of the selected species indicating a distinct expression pattern in different tissues among most species. Our study reveals that adaptive selection plays a key role in the evolution of PD1 and its ligands in the majority of vertebrate species, which is in agreement with the contribution of these residues to the mechanisms of pathogen identification and coevolution in the complexity and novelties of vertebrate immune systems.

## INTRODUCTION

The activation of mature peripheral B and T cells induces programmed cell death-1 (PD-1), a member of the CD28/CTLA-4 family [1, 2]. PD-1 and its ligands (PD-L1 and PD-L2) maintain peripheral tolerance by negatively regulating antigen receptor signaling [3]. PD-L1 is extensively distributed on non-lymphoid tissues, non-hematopoietic cells, leucocytes and pancreatic islets. PD-L2 expression is restricted to monocytes and dendritic cells (DCs) [4]. PD-L1 and PD-L2 ligation act as a secondary signal to T cells in combination with T-cell antigen receptor (TCR) signaling and results in the co-stimulation of a negative or inhibitory signal [5] that prevents the activation of TCR-mediated T cells and the production and proliferation of cytokines [6]. Recent studies have revealed that the PD-1/PD-L signaling pathway plays a significant role in autoimmunity and that the abnormal signaling of PD-1/PD-L results in the loss of peripheral tolerance and autoimmune disorders [7]. The expression of PD-L1 is observed on DCs, mast cells, T cells, B cells, macrophages, and nonhematopoietic cells, including astrocytes, vascular endothelial cells, keratinocytes, pancreatic islet cells, and corneal endothelial and epithelial cells. Both PD-L1 and PD-L2 are also expressed on tumor stroma and tumor cells. The appearance of PD-L2 at tumor positions may contribute to T-cell restriction mediated by PD-1 [8].

Both PD-1 and PD-L1 belong to the immunoglobulin (Ig) superfamily and are type I transmembrane proteins. PD-1 contains cytoplasmic domains that possess two tyrosine signal motifs, a transmembrane domain, and an Ig-V-like extracellular domain [2]. PD-L1 comprises a cytoplasmic domain that does not consist of recognized signaling motifs, a transmembrane domain, and Ig-V-like and Ig-C-like extracellular domains [9, 10]. Communication between the extracellular domains of PD-1 and PD-L1 promotes a conformational modification in PD-1, which results in the phosphorylation of the tyrosine-based immunoreceptor switch motif (ITSM) and the cytoplasmic immunoreceptor inhibitory motif (ITIM) by Src kinases [11]. Furthermore, the interaction of PD-1 and PD-L1 can also affect CD80, which may transport inhibitory signals to activated T cells [12]. The activation of PD-1 by PD-L1 changes T-cell activities in various ways, such as cytokine production, survival, the inhibition of T-cell propagation, and the functions of other effectors [13].

The interaction of PD-1 with PD-L1 is an important factor in immune tolerance. Mice lacking PD-1 are susceptible to developing lethal autoimmune cardiomyopathy or lupus-like autoimmune problems due to altered thymic T-cell responses [14, 15], and PD-L1 blockage has been shown to weaken feto-maternal tolerance [16]. These studies reveal an important role of PD-1 and its ligands in immune tolerance at the cellular and molecular levels [17]. It has been revealed that the expression of PD-1 in newly triggered T cells is involved in monitoring the strength of the early T-cell response upon the detection of an antigen [18]. In agreement with the role of PD-1 in modulating T-cell activities, the interruption of the PD1-PD-L1 interaction enhances the immune response toward various pathogens [19, 20].

Additionally, in some invertebrate clades, the genes directly interacting with pathogens and receptor genes are the most frequent targets of positive selection [21, 22]. Early comparative genomic studies recognized immune system processes as mutual goals of natural selection in Drosophila, mammals, primates, ants, bees, and other organisms [23–25] that harbored candidate signatures of selection. This suggests that pathogens, which evoke an immune reaction, maybe potent and steady selection pressures across species [26]. Previous studies on mammals revealed that proteins that interact with pathogens undergo twice as many amino acid variations as proteins that do not [27], and proteins that interact with Plasmodium undergo relatively higher adaptation rates [28]. In the context of conservation genetics, variation analyses at functional genomic regions provide an enhanced understanding of the mechanisms by which inbreeding and population bottlenecks can influence the adaptive potential of endangered species [29]. Early genome-wide studies have focused on single taxonomic ancestries (e.g., mammals) or restricted subgroups of candidate genes across lineages, but the accessibility of several new genomes now permits thorough evaluations across extremely diverse clades to estimate the extent to which specific genes show conserved regions of positive selection over extended evolutionary periods [25]. However, the role of selective pressure in natural populations in driving the diversification of additional aspects (non-MHC) of the immune system, such as innate immunity, remains poorly understood [30, 31]. Evidence has revealed that pathogens are the main selective pressure that drive evolution, and several new genomes now permit comparisons among various lineages to identify the extent to which particular genes undergo positive selection. The purpose of this study is to analyze the genomic sequences of programmed cell death-1 and its ligands in vertebrate species to calculate the selection pressure on these genes, which can contribute to adaptive evolution. Here, we explore the evolutionary routes by thoroughly analyzing these genes from a set of diverse vertebrates, and confer the role of selection and diversification of this gene family. Our results revealed that positive selection acting on PD1, PD-L1 and PD-L2 genes drives adaptive changes for biological functions directly related to immunological tolerance in vertebrates. The analysis in this study is likely to deliver understandings into the functional inference of the gene in the development of vertebrate evolution.

## RESULTS

This study was designed to analyze the genomic sequences of programmed cell death-1 and its ligands in vertebrate species to calculate the selection pressure on these genes, which can contribute to adaptive evolution. Our maximum-likelihood phylogenetic analyses of amino acid sequences from 166 vertebrate species revealed that PD1 genes evolved in a shared ancestor of vertebrates. In our study, we selected three genes (PD1, PD-L1, and PD-L2) that are involved in immune tolerance in vertebrates, and we also explored the evolutionary processes, phylogenetic relationships, and resulting structural and functional characteristics of PD1, PD-L1 and PD-L2 homologs in vertebrates. We analyzed the CDS of the PD1, PD-L1, and PD-L2 genes. We identified the orthologs of human PD1, PD-L1, and PD-L2 through a comprehensive BLASTP search approach. We filtered 202, 179 and 181 orthologs for PD1, PD-L1 and PD-L2 genes to retrieve the sequences in vertebrate species. The sequences were omitted if absent in most of the species or absent in two of the three of the main taxonomic groups and had sequences in less than 10 taxa. Secondly, poorly aligned sequences were screened using a sliding-window comparison method. These CDS were compiled into a complete multiple sequence alignment, which was used as the input for both the construction of Bayesian phylogenetic trees and other subsequent analyses.

### Protein domain analysis

**Figure 1:**
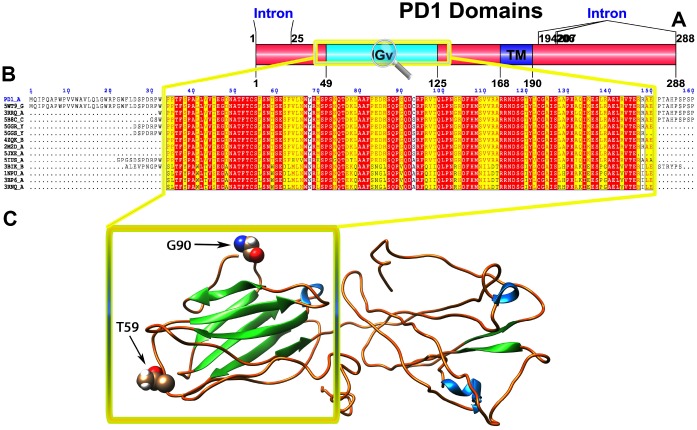
(**A**) Molecular structure of PD1 and Conserved domain analysis of PD1 protein. (**B**) Showing the MSA of the 20 most homologous proteins to PD1 (obtained with a BLAST+ search against the PDBAA database). Known secondary structure elements are displayed for all aligned sequences. Alternate residues are highlighted by gray. Identical and similar residues are boxed in red and yellow, respectively. (**C**) Location of positively selected amino acid sites identified PD1 conserved Ig domain. The crystal structure of human PD1 was used as a reference sequence and positively selected sites were drawn onto the crystal structure using Phyre tool (http://www.sbg.bio.ic.ac.uk/phyre2/html). Two residues identified under selection fall in the immunoglobulin-like domain containing the ligand-binding site. The sites which fall in the region identified as the ligand-binding site and another cluster in a region immediately following the signal sequence.

**Figure 2:**
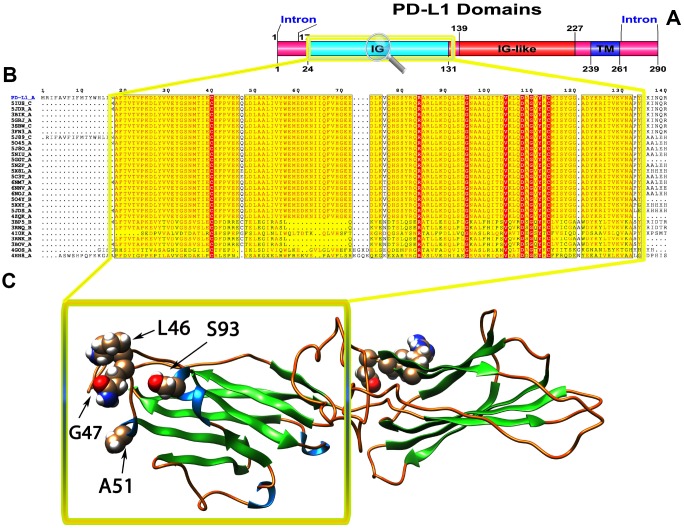
(**A**) Molecular structure and Conserved domain analysis of PD-L1 protein. (**B**) Showing the MSA of the 20 most homologous proteins to PD-L1 (obtained with a BLAST+ search against the PDBAA database). Known secondary structure elements are displayed for all aligned sequences. Alternate residues are highlighted by gray. Identical and similar residues are boxed in red and yellow, respectively. (**C**) Location of positively selected amino acid sites identified PD-L1 conserved Ig domain. The crystal structure of human PD-L1 was used as a reference sequence and positively selected sites were drawn onto the crystal structure using Phyre tool (http://www.sbg.bio.ic.ac.uk/ phyre2/html). Four residues identified under selection fall in the immunoglobulin-like domain containing the ligand-binding site. The sites which fall in the region identified as the ligand-binding site and another cluster in a region immediately following the signal sequence.

**Figure 3:**
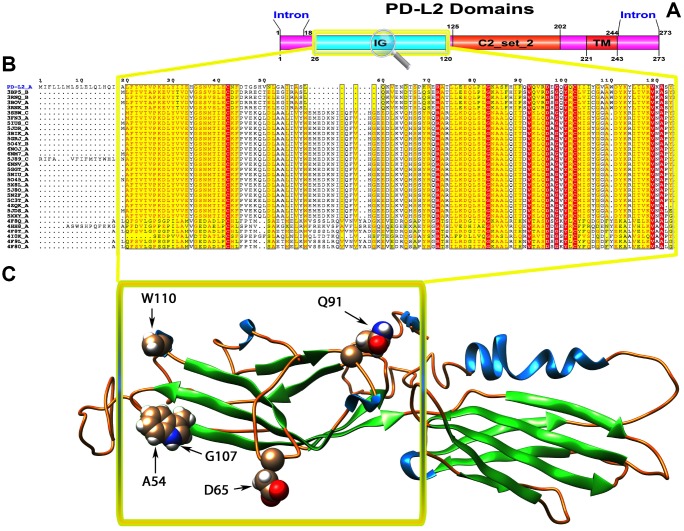
(**A**) Molecular structure of PD1 and Conserved domain analysis of PD-L2 protein. (**B**) Showing the MSA of the 20 most homologous proteins to PD-L2 (obtained with a BLAST+ search against the PDBAA database). Known secondary structure elements are displayed for all aligned sequences. Alternate residues are highlighted by gray. Identical and similar residues are boxed in red and yellow, respectively. (**C**) Location of positively selected amino acid sites identified PD-L2 conserved Ig domain. The crystal structure of human PD-L2 was used as a reference sequence and positively selected sites were drawn onto the crystal structure using Phyre tool (http://www.sbg.bio.ic.ac.uk/ phyre2/html). Five residues identified under selection fall in the immunoglobulin-like domain containing the ligand-binding site. The sites which fall in the region identified as the ligand-binding site and another cluster in a region immediately following the signal sequence.

To recognize the domains of these proteins, we used the SMART online tool to predict the protein domains. We identified two domains (Ig-V and TM) in PD1 (Figure 1A), three domains (Ig, Ig-like and TM) in PD-L1 (Figure 2A) and three domains (Ig, C2_set-2 and TM) in PD-L2 (Figure 3A). According to the literature, Ig-like domains are present in various protein families, which are related both in structure and sequence. Ig-like domains contribute to various functions, such as the immune system, cell surface receptors, cell recognition, and muscle structure formation [32]. The V-set domains are Ig-like and resemble the antibody variable region. The V-set domains are present in various protein families, including Ig heavy and light chains; T-cell receptors, such as the cluster of differentiation (CD2, CD4, CD80, and CD86); tyrosine-protein kinase receptors; myelin membrane adhesion molecules; and junction adhesion molecules (JAM) in PD1 [33]. C2-set domains are Ig-like domains that resemble the antibody constant domain and are mainly found in the mammalian T-cell surface antigens CD2, CD4 and CD80 and in intercellular and vascular cell adhesion molecules (ICAMs and VCAMs) [34].

### Positive selection analysis

We used different site models to recognize the genes under positive selection across the vertebrate species. We compared different models for the selected genes in the data set using the phylogenetic tree as input data. We performed a likelihood analysis that compared different models based on ω ratios to identify the codons under positive selection in the corresponding genes. These likelihood models included an additional ω parameter for some fraction of sites and models that do not include the additional ω parameter. The codeml program was used to compute the parameters related to gene selection among 55 species, and a positive selection test was analyzed by the two models for M1a and M2a and for M7 and M8. The results indicated that the test of the PD1 gene in M1a-M2a was not significant with the likelihood test value 2ΔlnL=0 (p>0.05), while in M7-M8, the likelihood ratio test (2ΔlnL) was 5.103. The test results of positive selection model M8 were significant (p<0.05); the PD1 gene was under positive selection indicating that the M8 was accepted, while M7 was rejected. In the PD-L1 gene, the codon sites were positively selected with P>95% and P>99%, based on the Naïve Bayesian (naïve empirical method based on Bayes, NEB) and Bias (Bayes empirical Bayes experience method, BEB) analysis. However, for the PD-L2 gene, both sets of models M1a: M2a and M7: M8 was highly significant, with 2ΔlnL values of 137.60 and 827.14, respectively (Table 1). We evaluated the global ω values to further conclude the evolutionary signatures of positive selection by using MEME, FEL, and SLAC analyses. Our results revealed robust evidence of positive evolutionary selection for the PD1, PD-L1 and PD-L2 genes in the vertebrates. We applied the Bayesian method to identify the sites under selective pressure by calculating the posterior probabilities for each codon. The sites with higher probabilities are more likely to be under positive selection with ω>1 than sites with lower probabilities. We identified several sites under positive selection in the PD1 protein by using BEB analysis, with most sites exhibiting high posterior probabilities at 99% or 95%. To evaluate the false positive outcomes of PAML, we further confirmed the positive selection using the Selecton server that recognizes adaptive selection at individual amino acid sites in the protein. The MEC model identifies the variations among amino acid exchange rates. As a result, we identified an adaptive selection at various amino acid sites in PD1 (Supplementary Figure 1), PD-L1 (Supplementary Figure 2), and PD-L2 (Supplementary Figure 3). In our analysis, we found that there were few sites in the Ig-V-like domain region in these proteins, and this Ig-V-like domain had evolved. However, the proteins that experience positive selection might be conserved and exposed to purifying selection during adaptive evolution. This conservation derived from the evolution of each amino acid/nucleic acid position was identified by using the ConSurf server. The color-based representation of the ConSurf server allows the identification of regions with strong and weak conservation in the structure of the PD1, PD-L1 and PD-L2 proteins (Figures 1B, 2B and 3B, respectively). We found that several conserved residues had masked the signals of selection, and the residues that were buried or exposed according to the neural network algorithm were determined to be under positive selection on variable positions in the PD1, PD-L1 and PD-L2 proteins (Supplementary Figures 4–6, respectively).

**Table 1:** Log-Likelihood Values and Test Statistics for PAML Site Models of positive selection.

**Gene**	**Models**	**Parameter estimates**	***lnL***	***LRTs***	**Positively selected sites PAML**	**SLAC**	**FEL**	**MEME**
PD1	M1a	p1: 0.51480 p2: 0.48520	-23577.93	0	0	81, 471, 594	79,81,82,90,107,141,23 6,245,255,260,271,293, 300,301,303,305,306,30 7,309,310,316,218,322, 323,324,325,326,333,33 5,337,342,347,353,353, 357,359,362,364,436,45 8,471,498,594,607,615	81,108,109,110,24 4,411,412,459,471 ,495,505,594,595,
		ω1: 0.21574 ω2: 1.00000						
	M2a	p0: 0.51480 p1: 0.39898 p2: 0.08622	-23577.93					
		ω0: 0.21574 ω1: 1.00000 ω2: 1.00000						
	M7	P: 0.98018 q: 1.49822	-23414.34	5.103112*				
	M8	p0: 0.96818 p: 1.00330 q: 1.60757	-23411.79					
		p1: 0.03182) ω: 8.05118						
PD-L1	M1a	p1: 0.44199 p2: 0.55801	-21407.94	137.60***	96**, 143, 292*, 293**,294**, 332**,354*, 553**,555**, 560**, 614**, 615**	293,294,332,553,614	45,53,55,62,64,65,66,67 ,69,70,71,73,76,77,78,8 0,81,82,84,85,87,88,97, 98,100,112,114,115,116 ,124,142,152,153,154,1 55,162,166,168,188,201 ,206,213,239,353,615,6 17	96,110,111,292,33 2,397,550,553,554 ,555,614,000
		ω1: 0.18081 ω2: 1.00000						
	M2a	p1: 0.39702 p2: 0.47651 p3: 0.12647	-21339.13					
		ω0: 0.18153 ω1: 1.00000 ω2: 3.26240						
	M7	P:0.66997 q: 0.64572	-21305.29	131.62***				
	M8	p0: 0.86159 p: 0.77177 q: 0.94085	-21239.48					
		(p1: 0.13841) ω: 2.57746						
PD-L2	M1a	p1: 0.48081 p2: 0.51919	-22727.73	137.60***		132,134,137,138,139, 141,146,148,152,153, 154,155,162,194,207, 227,230,231,234,238, 293,294,318,320,350, 436	97,103,122,132,134,135 ,137,139,145,146,148,1 55,156,167,172,173,194 ,207,226,227,231,232,2 34,238,239,240,253,269 ,270,289,290,293,294,2 95,318,320,321,332,336 ,339,343,350,407,417,4 36,554,562,608,	89,122,278,305,35 4,407,561,595,597 ,604,608,000,613, 614,621,
		ω1: 0.20239 ω2: 1.00000			54**, 55**, 59**, 62**, 80**, 81**, 82**,83**, 84**, 85**,597**,598**, 599**,600**,608**			
	M2a	p0: 0.35589 p1: 0.38002 p2: 0.26409	-22297.1					
		ω0: 0.20533 ω1: 1.00000 ω2: 9.06683						
	M7	P:0.56058 q: 0.42402	-22649.48	827.14***				
	M8	p0: 0.73476 p: 0.70098 q: 0.70029	-22235.91					
		(p1: 0.26524) ω: 7.39740						

The proportion of sites under positive selection (*p*1), or under selective constraint (*p*0), and parameters p and q for the beta distribution. Parameters indicating positive selection are in bold. *p*: significant at 5% level; p: significant at 1% level. Sites potentially under positive selection identified under model M8 are listed according to the human sequence numbering. Positively selected sites with posterior probability .0.9 are underlined, 0.8–0.9 in bold, and 0.5– 0.7 in plain text. The test statistic *2Δl* is compared to a χ2 distribution with 2 degrees of freedom, critical values 5.99, 9.21, and 13.82 at 5%, 1%, and 0.1% significance, respectively. **: significant at 1% level; *: significant at 5% level.

**Figure 4:**
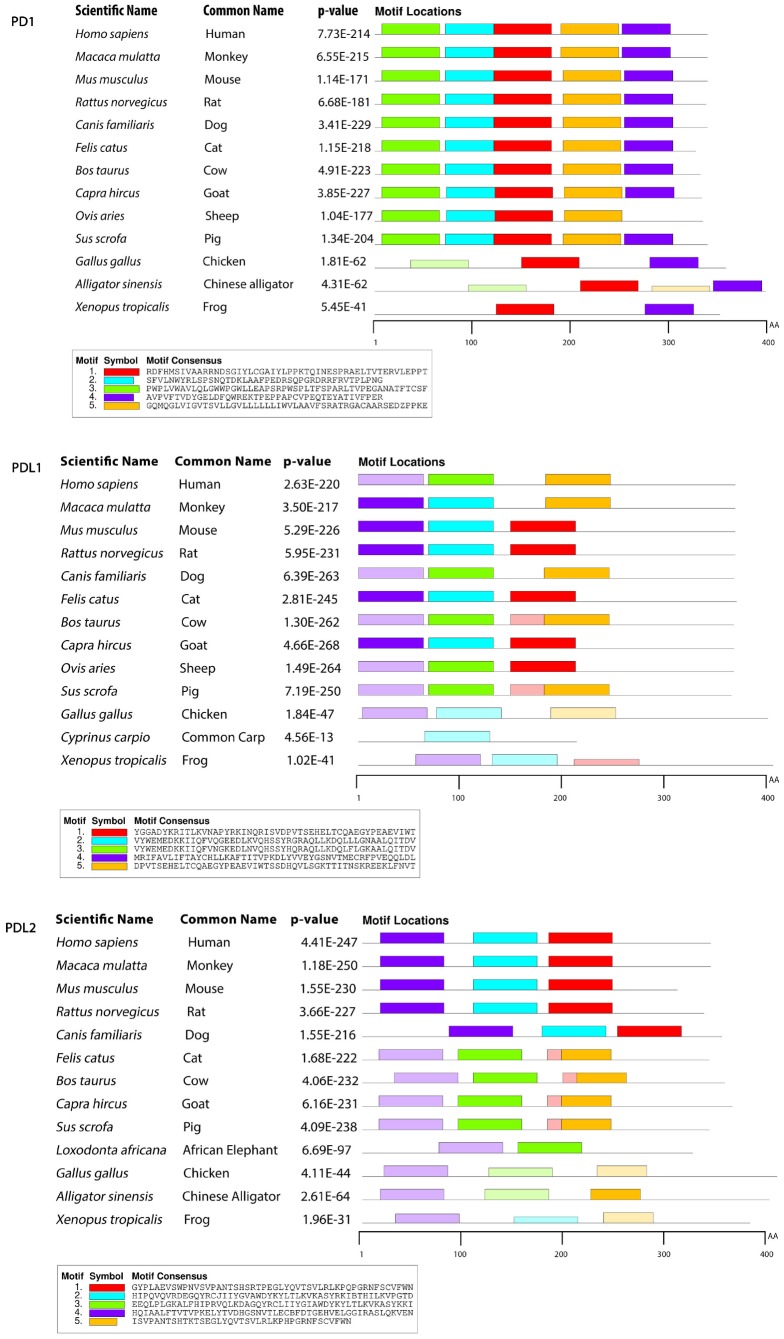
**Motif distribution of PD1, PD-L1 and PD-L2 genes in representative vertebrate species.** Motifs of these genes from representative species from each group are predicted using MEME suite (http://meme-suite.org/) based on amino acid sequences. All sequences are separated by 5 conservative motifs with colors, including motif 1 (red), motif 2 (cyan), motif 3 (green), motif 4 (purple) and motif 5 (brown).

To gain insight into the probable intermolecular interactions of these positively selected regions of the PD1 proteins with conserved functional domains, we generated 3D models of the proteins with a reported complex between the Ig region and the protein-coding region, which is the target of Ig-like proteins, as a models for homology modeling, assuming that this conserved Ig region could interact correspondingly with its targets. The 3D protein structure showed that T59 and G90 were the main PD1 protein-protein interaction residues under positive selection (Figure 1C), and L46, G47, A51 and S93 were the main interacting residues that were detected under strong selective pressure in the PD-L1 protein (Figure 2C). The residues A54, D65, Q91, G107 and W110 were the main interacting residues found under selection in the PD-L2 protein (Figure 3C). Motif analysis by MEME identified various species in our data set that shared high conservation in motifs 1 to 5 but differed in motif 1, which we determined was lacking in the PD1 protein of birds (Figure 4 and Supplementary Figure 7). Within the same subfamily, individuals had similar motif distributions, such as PD-L1 and PD-L2 lacking motif 5 in both avian and amphibian species, demonstrating that individuals of the same subfamily may have similar functions. All motifs were found in all protein sequences from diverse vertebrate species, excluding some of the mammalian species, including *Chlorocebus sabaeus*, which lacks motif 1 and motif 5 in PD1, *Monodelphis domestica*, which lacks motif 5 in PD1, and *Gorilla gorilla* and *Castor canadensis*, which lack motif 5 in PD-L1 (Figure 4 and Supplementary Figure 8). Different motif patterns were observed in PD-L2, where motif 5 or motifs 2-5 were missing in *Mus musculus*, *Canis familiaris* and *Oryctolagus cuniculus* (Figure 4 and Supplementary Figure 9). The lack of motifs in various species signifies the divergence of gene structural features concerning exon-intron relationships. These analyses revealed that the differences in motif distribution in PD proteins of vertebrate species might have diverged from the functions of these genes during adaptive evolution.

### Lineage-specific selection analysis

The codon-based selection model can only classically confer positive selection signals when particular codons are under selection pressure in several lineages. We used an adaptive branch-site random effects likelihood (aBS-REL) model to relax this hypothesis to calculate the selection probability and identify selection restricted to specific lineages independently at each subgroup of the phylogeny. To further validate our site-model results, we used aBS-REL for each gene to identify the lineages that underwent positive selection during adaptive evolution. We noticed that the genes recognized as being under positive selection by BUSTED across mammalian lineages were also under significant positive selection in additional lineages according to the aBS-REL model (Figure 9; Supplementary Tables 1–3). Clades within avian, mammalian, and reptilian lineages showing considerable signals for positive selection (p<.05) were identified using the branch-site-REL (BSR) program executed in the Data Monkey Web Server. PD1 exhibited strong signatures of positive selection at various nodes of its mammalian and avian clades, including *C. sabaeus, Rhinopithecus roxellana, Acinonyx jubatus, Ovis aries* and *Meleagris gallopavo*, respectively (Figure 9). PD-L1 showed significant selection within a broad range of phylogeny. Positive selection signals for PD-L1 were identified at several nodes in two major clades: *O. aries*, *Pantholops hodgsonii*, *Sus scrofa, C. sabaeus, Macaca fascicularis, C. canadensis,* and *M. domestica* in the mammalian clade and *Apteryx australis mantelli, Falco peregrinus, Coturnix japonica, Taeniopygia guttata,* and *G. gallus* in the avian clade (Figure 9). However, for PD-L2, we obtained surprising results: all clades in the dataset showed strong signals of positive selection in vertebrate lineages (Figure 9).

### Gene enrichment analysis

**Figure 5:**
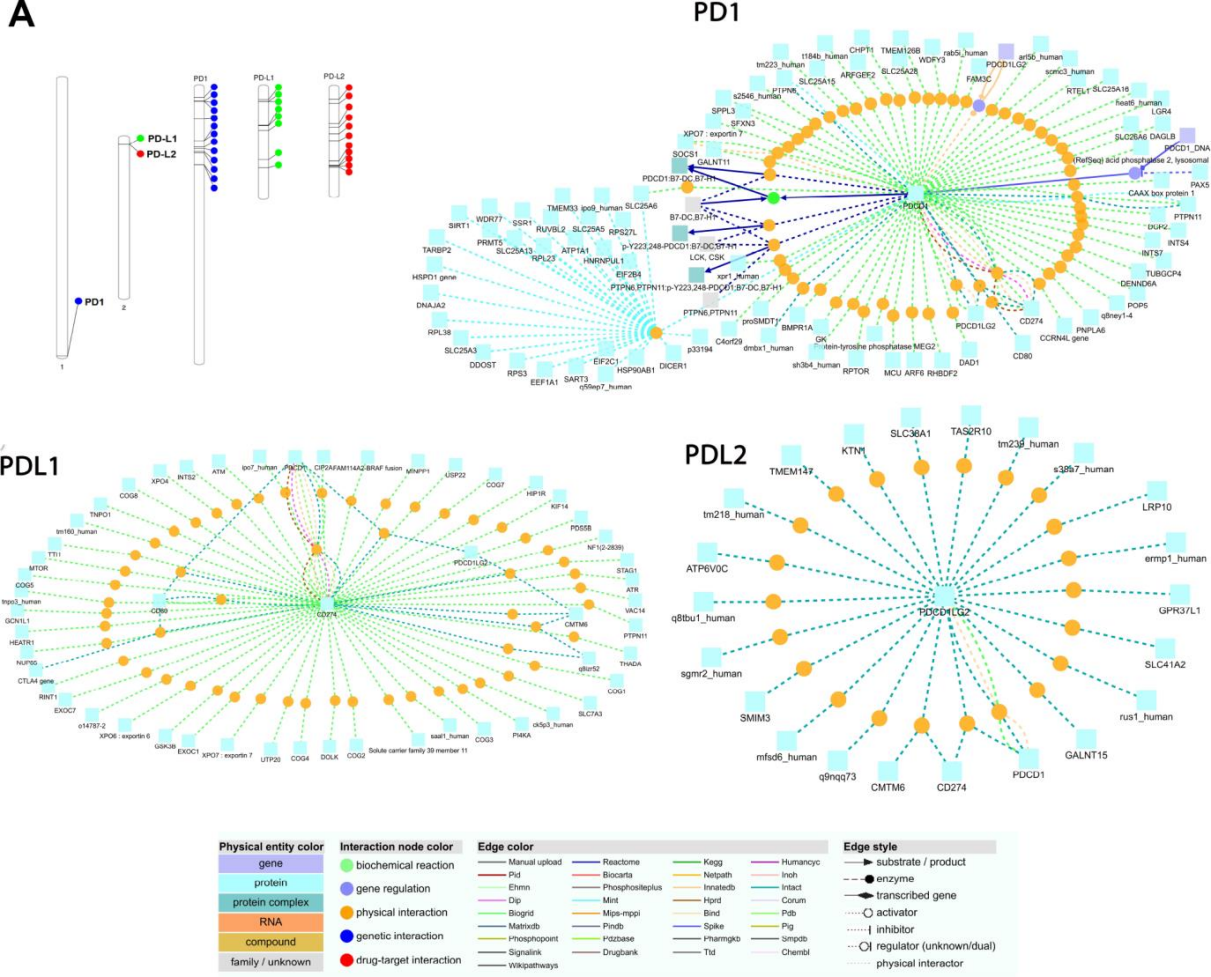
(**A**) Chromosomal locations and positively selected sites of PD1, PDL1, and PDL2 genes. The chromosome number is indicated above each bar. The chromosome size is indicated by its relative length using the information from NCBI. The scale of the chromosome is millions of base pairs (Mb). Functional interaction network of PD1, PDL1 and PDL2 genes generated by the visualization environment of Consensus Path DB meta-database, after conserved synteny and functional enrichment analysis. The network of the PD1 gene contains 107 interactions and 62 physical entity nodes. The network of PDL1 contains 125 interaction and 55 physical entity nodes. The network of PDL2 contains 56 interaction and 22 physical entity nodes. Each node represents a physical entity (gene, protein or compound). Each edge represents an interaction.

We used EnrichNet, which is a network-based gene enrichment analysis program, and gene IDs from the Ensembl database (PD1; ENSG00000188389, PD-L1; ENSG00000120217, and PD-L2; ENSG00000197646) as queries for the functional network analysis. We used the immune system regulation activity class of the Gene Ontology (GO) functional catalog to map the synteny genes and obtained networks including PD1, PD-L1, and PD-L2 as functionally associated genes (Figure 5). We selected the regulation of the immune response group because it includes the PD1, PD-L1 and PD-L2 genes in most of the functional databases. We expanded our enrichment analysis to various databases and all functional classes by generating functionally linked gene networks in the ConsensusPathDB obtained from the EnrichNet analysis. Using this technique, we identified an interaction network with 107 interactions and 62 unique nodes in the PD1 protein (Figure 5). The interaction between PDCD1 and PTPN6 was maintained through a transcription factor encoded by PTPN, which is the py223 gene that interacts with the PTPN11 and dmbx1 genes (Figure 5). We found 125 interactions and 55 unique nodes physically interacting with the PD-L1 protein (Figure 5). PD-L2 was identified in the conserved synteny map with 56 interactions and 22 distinct nodes, but its function was associated with KTN1, GALNT15 and TMEM147 after the enrichment analysis (Figure 5).

### Bioinformatics analysis

**Figure 6:**
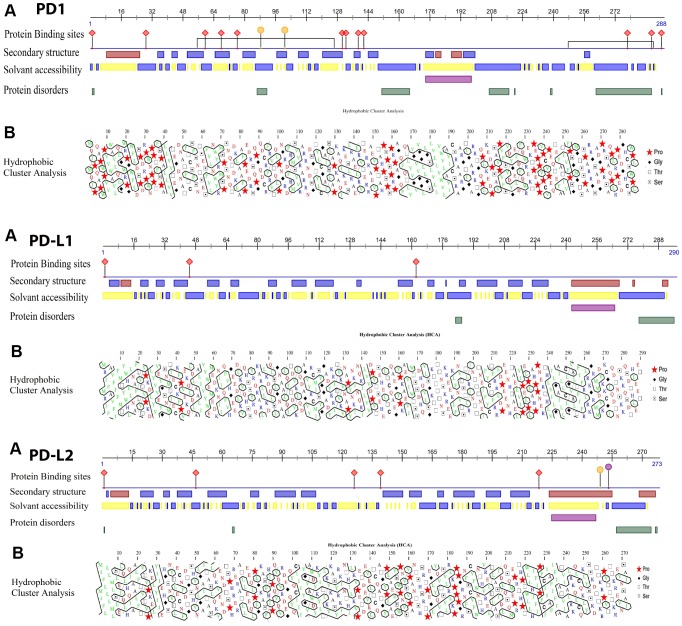
(**A**) Proteins analysis showing the results of the binding site, solvent accessibility and protein disorder predictions in the human PD1, PD-L1 and PD-L2 sequences. (**B**) Hydrophobic cluster analysis (HCA) plots of the human PD proteins. HCA plots were constructed with the HCA 1.0.2 program. HCA uses the standard one-letter amino acid abbreviations except for four amino acids, as shown in the key. Hydrophobic residues are outlined. Clusters of hydrophobic residues are usually associated with regular secondary structures (α helices or β sheets). Zigzagging vertical lines of hydrophobic residues indicate alternating hydrophobic and non-hydrophobic residues, typical of exposed β sheets (for example, β2, β3, β5, and β6). Continuous hydrophobic clusters are more common in internal β sheets.

Homologous sequence analysis of PD1, PD-L1 and PD-L2 across several clades was used to predict related structural topographies of these proteins. The structure of the human PD1, PD-L1 and PD-L2 genes were used as a model for further analyses. In the case of PD1, there was higher variation among the homologous sequences than among the sequences corresponding to various domain regions. The prediction of solvent accessibility, coils and turns in the corresponding domain region (Figure 6A), regardless of hydrophobic clustering development, suggested that protein-protein interfaces were developed by the contribution of specific parts of this region, which are important for the interaction of PD1 with its targets. The possible development of these clusters was revealed by HCA in regions consistent with the Ig-like conserved domain (Figure 6B). The secondary structure prediction results display numerous helices and a reduced number of strands in the PD-L1 and PD-L2 protein sequences. Some of these secondary structures were confined within the conserved regions of PD-L1 and PD-L2, which supports the hypothesis that β-strands and helices are the most rigid types of secondary structures and that mutations might disrupt the secondary structure of proteins.

### Coevolution analysis

**Figure 7:**
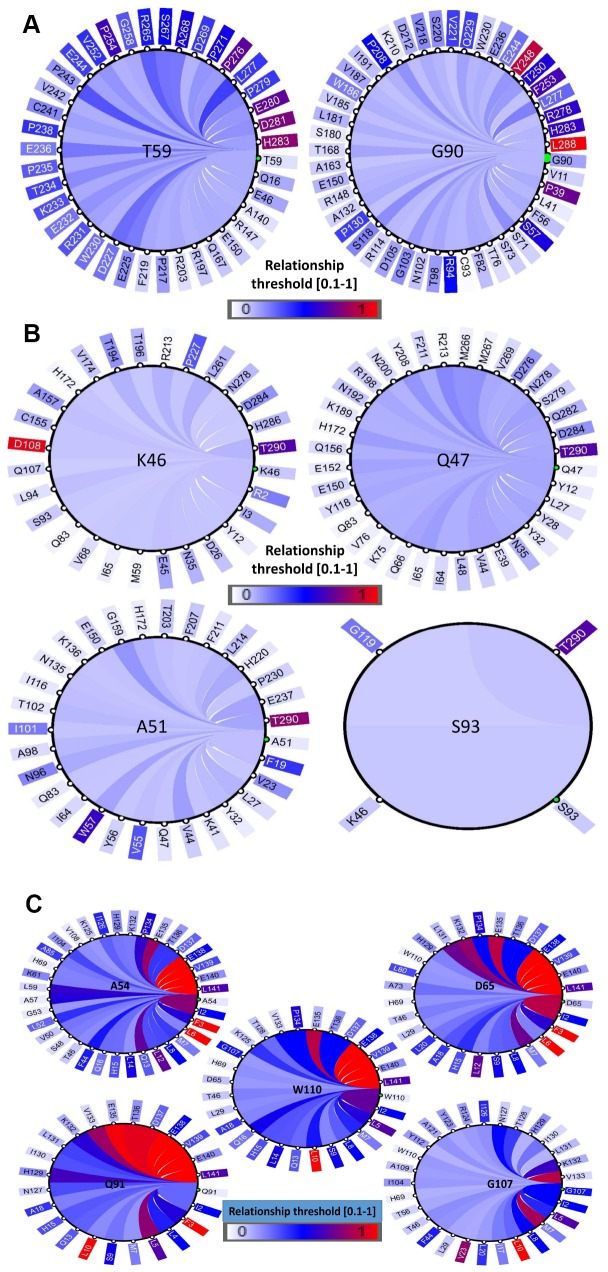
**Coevolution analysis of positively selected conserved domain residues.** The circular relation diagram centered on the residues with their top co-varying residues at cutoffs (**A**) PD1, (**B**) PD-L1 and (**C**) PD-L2. Labels on the diagram represent amino acid residues and their positions in the protein sequence. Colors of the arcs represent covariance scores between two given positions. Colors of the arcs represent covariance scores between two given positions.

The structural and functional features of the positively selected residues were further examined through co-evolution analysis by identifying the coordinated contacts among these residues. This was performed by the identification of other residues that have co-varied with positively selected residues during evolution. This coevolving relationship among various amino acid sites within a protein could be the result of their structural or functional interactions. Hence, we conducted a coevolution analysis using homologs of PD1, PD-L1 and PD-L2 as inputs and identified various coevolving residue pairs that were identified as being under positive selection in the former analyses. A diagram showing the networks was built to recognize a link of significantly associated residues (Figure 7A–7C). We have identified that amino acids with a greater number of co-evolutionary contacts likely evolve more steadily than those with fewer co-evolutionary contacts. We also determined that the residues with strong connectivity in the network, such as Y248 and L288 in PD1, D108 and T290 in PD-L1, and F3, L6, and L10 in PD-L2, were the residues with higher conservation, respectively (Figure 7A–7C). The positively selected residues were present in the nodes of a subnetwork that was constrained to amino acid residues present in the conserved Ig-like and Ig-V-like domains in the protein. Multidimensional scaling (MDS) scatterplots of co-varying residues in human PD1, PD-L1 and PD-L2 exhibited coevolving probabilities according to Pearson correlation ®. From this covariance analysis, we determined the distances and contacts of positively selected residues in the protein domains and clusters of functionally related residues (Supplementary Figure 10). The 3D viewing pane provides interactive zoom and rotation capabilities and labels selected residues. These outcomes support the proposal that the PD protein regions conforming to these conserved domains are structural-functional units.

### Protein structure quality analysis

These positively selected residues were present in the loop, which can dock into the Ig-binding pocket. The extent of conservation of these amino acids was diverse, with only W110 and E136 being comparatively conserved in PD-L2 (Supplementary Figure 6). The low degree of conservation among the other residues can be conferred to neutral variations along with these sites according to the positive selection/purifying analysis (Supplementary Figures 1–3). Moreover, the complete quality of the predicted structures was evaluated by ERRAT, ProSA and VERIFY3D values. The Ramachandran plots were used to check the backbone conformation angles for the respective residue in the modelled protein crystal structures, which showed the empirical scattering and calculated energies of the residue coordinate system to display either plot of the conformation detected in the databank of identified 3D models or outlines or steric measures as a function. We observed that most of the residues were found in the allowed region of the graph that represented the conformational accuracy of all predicted models. For PD1, PD-L1 and PD-L2, the number of residues in the favored area was 95.9, 96.5, and 95.9%; the number of residues in the allowed region was 3.6%, 3.2%, and 3.6%; and the number of residues in the outlier region was 0.5% for all predicted structures, respectively (Supplementary Figure 11). The z-values show the complete model excellence of the predicted structures with values of -7.2, -7.8, and -6.7, respectively (Supplementary Figure 11). Additionally, the compatibility of all predicted structures and their correctness was validated using the VERIFY3D software package. The dynamics of the PD1, PD-L1 and PD-L2 protein model structures were computed using the Gaussian network model (GNM), which revealed that the residues that showed greater mobility were part of the binding pocket and could function as the protein-protein interaction region. The cross-correlation plots represented the variation in residues and their physical behavior. The predicted plot outcomes were analyzed based on the colors blue, dark red, yellow and cyan. We identified that the entirely correlated pairs were shown in dark red, whereas the anti-correlated pairs are were shown in blue. Additionally, the uncorrelated and moderately correlated regions were colored cyan and yellow, respectively (Supplementary Figure 11).

### mRNA expression of the PD1, PD-L1 and PD-L2 genes

**Figure 8:**
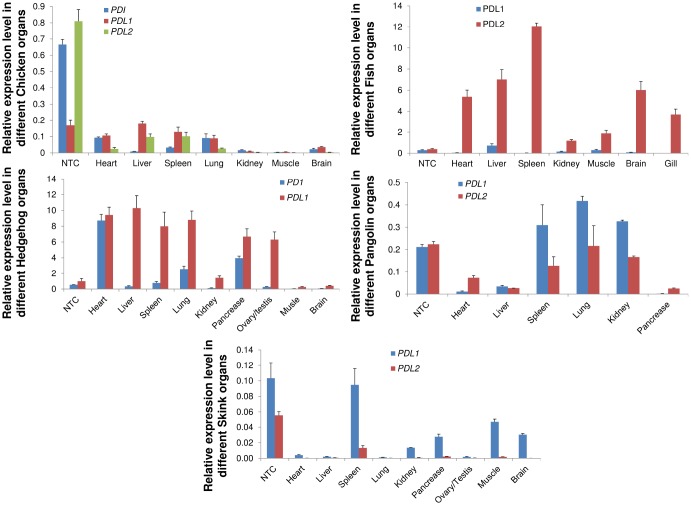
**qRT-PCR analysis of PD1, PD-L1 and PD-L2 genes in different animal tissues.** Expression patterns of genes in different tissues were examined. Heart, liver, spleen, lungs, kidney, pancreas, brain, were used for quantitative reverse transcription (qRT-PCR) polymerase chain reaction. Transcript levels are expressed relative to that of beta-actin. NTC: negative control.

We investigated the mRNA expression patterns of the PD1, PD-L1 and PD-L2 genes in five vertebrate species, including *M. javanica, A. albiventris, G. gallus, A. schrenckii* and *S. crocodilurus*. We examined the mRNA expression profiles in heart, liver, spleen, lung, kidney, pancreas, brain, testis, and ovary and muscle tissues from male and female adults. We noticed that PD-L1 had increased expression in almost all species and that PD1 exhibited expression only in chicken tissues. PD-L2 showed increased expression in pangolin and fish in almost all tissues with some exceptions (Figure 8). The expression of PD-L1 was high in almost all tissues, indicating tissue-specific expression. The PD1, PD-L1 and PD-L2 genes were highly expressed in the heart, liver and spleen, indicating their predicted functions in immune development and the prevention of autoimmune disorders in organisms. These PD genes that were highly expressed in vertebrate tissues or organs may have important functions in signaling pathways that play a significant role in autoimmunity, and abnormal signaling in these pathways may result in the loss of peripheral tolerance. Hence, the tissue specificity of the PD genes identified here may be useful sources for further probing their biological functions in immune tolerance at the cellular and molecular levels.

## DISCUSSION

Key evolutionary changes occur in the genomes of animals via divergence, the assimilation of genetic data from independent lineages, gene duplication, and epigenesis. Horizontal gene transfer and genome duplications lay the foundations for all main molecular mechanisms of adaptive immunity [35]. Previous studies on the evolution of immune genes in birds mainly focused on the coevolution of host-pathogen hotspots, including the MHC and TLRs [36, 37]. The positive selection signatures we identified were consistent with the adaptive selection here. PD-L1, PD1 and PD-L2 across vertebrate genes are first involved in regulating lymphocyte activation, immune system function, the promotion of T regulatory cell function and development, the progression of autoimmunity and immune tolerance as targets of positive selection. We identified numerous positions in three genes under positive selection in mammals, reptiles and birds (Table 1). We found that the genes with significantly increased overall distinctive expression in these lineages resulted in standards paralleled to those not under selection pressure or positively selected only in mammals. Across vertebrate clades, our results indicate that pathogens might be a stable selective pressure. Previous phylogenetic studies revealed uncertainties related to the first vertebrate phylogeny change in IRF, i.e., where we find an adaptive immune system. It was proposed that in the beginning, the IRF family divided into two branches that are found in all cnidarians and bilaterians [38]. Although facts and evidence continued to amass, the four antecedents probably advanced into the four classes of vertebrates, IRF1-G (IRF2IRF1), IRF4-G (IRF4, IRF9, IRF8, IRF10), IRFs, IRF3-G (IRF7, IRF3), and IRF5-G (IRF6, IRF5), following 2-fold whole-genome duplications [38, 39]. In the evolution of the PD proteins to know the comparative characters of positive selection, we used two approaches to compare the difference at the codon level: an evolutionary model (M8) that allowed positive selection, and another using the MCMC evolutionary model implemented in MrBayes implemented in the Selecton server [40, 41]. *ω* values for the individual site were calculated in both circumstances. Our consequences demonstrate the conservation of the Ig domain of PD-L1, PD1, and PD-L2 coding sequences attained after protein alignments MAFFT (Figures 2B, 1B, and 3B). The fallouts allowing for only the PD mature proteins have revealed that the normal *ω* values were 0.18153, 0.21574 and 0.20533 using M2 and M1 evolutionary models, correspondingly. These results revealed that the protein changing gradually in the regions under purifying selection undergo non-identical switches, which are detrimental to health and thus have low chances of fixation during evolution [42]. The subsequent process favored positive selection and identified the amino acid residues with a ω>1 (Table 1). Rendering cross-correlations analysis between filtrate variations, this movement is connected in the section of the protein chain, which protects amino acids L288 and Y248 in PD1, D108 and T290 in PD-L1, and, L6, F3 and L10, are the filtrates with higher management, correspondingly (Figure 7A–7C). When the study deliberated the area consistent to the N-terminal, three positively selected sites L6, F3 and L10, were found in PD-L2 using the M8 evolutionary model (Figure 3C) with an ordinary *dN/dS* value of 7.39740. Our outcomes reveal that several sites in other proteins, which are under substantial positive selection have been developing more swiftly than the mature protein [43–45]. As a result, the dynamic selection forces its change which concerns to improve the protein secretion efficacy, which is true in case of PD-L2 protein (Figure 3C), which is unlike than the matured protein [46, 47].

**Figure 9:**
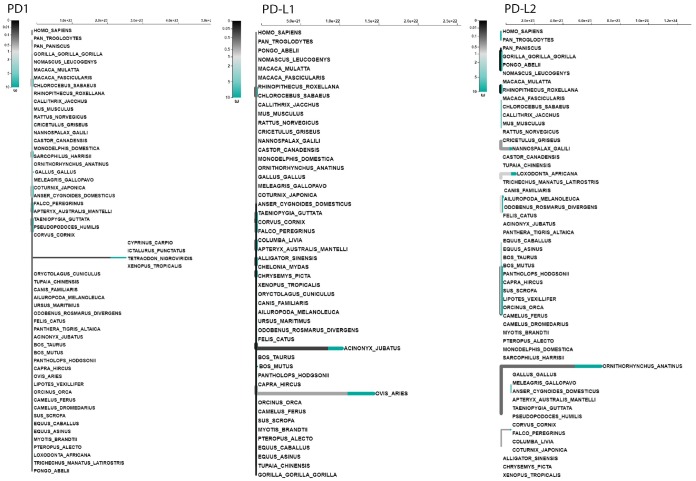
**Adaptive branch-site REL test for episodic diversifying selection in PD1, PD-L1 and PD-L2 genes.** The phylogenetic tree scaled on the expected number of substitutions/nucleotides. The hue of each color indicates the strength of selection, with primary red corresponding to ω > 5, primary blue to ω = 0 and grey to ω=1. The width of each color component represents the proportion of sites in the corresponding class. Thicker branches have been classified as undergoing episodic diversifying selection by the sequential likelihood ratio test at corrected ≤0.05.

We performed a branch-site test to determine the specific branches under selection in vertebrate clades, and we found few branches of mammalian clades under selection in the PD1 gene (Figure 9). In the case of PD-L1, positive selection was identified in mammalian and avian clades (Figure 9). However, surprising results were identified during the analysis of the PD-L2 gene, in which we found that there is positive selection in most of the branches of vertebrate clades in the data set. (Figure 9). Because branch-site analysis can lead to false implications of positive selection due to multi-nucleotide mutations, we further validated our results through the *aBS-REL* model, and similar patterns of selection were observed between the *aBS-REL* and site models. The results suggest that the overall selection patterns we observed were accurate with the alternate analysis. Generally, the PD1 protein in vertebrate lineages revealed no evidence of positive selection that suggested a constant selection pressure hampering genetic variation, mainly in the avian clade. Nearly a complete lack of positive selection, as observed by the M8 evolutionary model, has been entirely supported by *LRT* [48]. This may be associated with the concept that the evolutionary history of the PD1 gene has occurred without gene duplication events. Gene duplication is one of the evolutionary approaches that permit versatile advancement in genomes. It has been revealed for other proteins that positive selection happens after a duplication event that proposes an unwinding of the selective pressure supporting genetic variation [49, 50]. This relaxation was missing not only in avian but also in other vertebrates’ lineages during PD1 evolution, conferring to the Bayesian phylogenetic approaches (Supplementary Figure 10). Overall, these findings suggest that PD1 molecular evolution has classically been determined by purifying selection. The synteny of PD1, PD-L1, and PD-L2 is conserved among vertebrate clades, and the variations found in some motifs could be described by the varying evolutionary backgrounds of these genes for homologous arrangements in mammals and other vertebrates. A comparing quality set enrichment analysis shown a functional relationship between a subset of the conserved synteny genes, PTPN6, KTN1, GALNT15 and TMEM147 might be supported through the transcription factor, encoded by the PTPN; py223 gene (Figure 5). The purifying/positive selection investigation did not distinguish positive selection in amino acid residues of PD1, showing that purifying choice, especially within the regions comparing to Ig like domain has driven PD1 molecular evolution. A co-evolution examination showed that residues within the regions comparing to each motif especially co-vary with each other during PD1 evolution.

The structure and function of proteins are reliant on synchronized connections among their amino acid residues. Therefore, the identification of structural characteristics of positively selected amino acid residues could resemble the detected residues that co-vary with each other during evolution. This co-evolutionary relationship among amino acid residues within proteins could be the consequence of their structural or functional interactions. Previous studies on protein coevolution have discovered roles in protein constancy and intermolecular interactions [51–54]. Therefore, the variation in PD1 and its ligands may occur during evolution. Here, we found that Ser93 of human PD-L1 and Gly107 in PD-L2 may have important roles in some of the previously revealed functions by estimating the *dN/dS* of mammalian PD1 sequences. PD1, PD-L1, and PD-L2 have domains that are highly conserved among vertebrates, indicating that their roles are not redundant and that this selective pressure may derive from PD specificity for their immune system regulation. However, proteins that directly interact with other molecules tend to have an increased chance of adaptation to fit each other’s evolutionary changes. PD genes may have undergone such co-evolutionary traits in the context of their original function. Therefore, a coevolution analysis was performed via multiple sequence alignments generated for PD1, PD-L1 and PD-L2 homologs, and we found positively selected coevolving residues showing substantial variability (Figure 7). These results suggest that these regions corresponding to domains are structure-function modules within the PD proteins. The homology modelling of the PD protein regions corresponding to Ig-like domains and subsequent dynamics region was performed using a GNM (Supplementary Figure 11).

## CONCLUSIONS

PD1, PD-L1, and PD-L2 have Ig-like domains that are highly conserved among vertebrates, which may enhance the understanding of their role in biological systems such as immunological tolerance. Our results revealed that positive selection acting on PD1, PD-L1 and PD-L2 genes drives adaptive changes for biological functions directly related to immunological tolerance in vertebrates. Our maximum-likelihood phylogenetic analyses of amino acid sequences from 166 vertebrate species revealed that PD1 genes evolved in a shared ancestor of vertebrates. According to the results, the major evolutionary processes causing the sequence variation observed in PD1 in vertebrates were adaptation and selection. Future studies integrating molecular data and pathogenicity evidence will help to determine the selective forces behind the long-term adaptation of programmed cell death genes, as well as to determine the genetic conflicts between immune system development pathways and immune tolerance.

## MATERIALS AND METHODS

### Ethics statement

This study was approved by the Ethics Board for Animal Trials at the Guangdong Institute of Applied Biological Resources (reference number: GIABR20170720) by following the basic ethical guidelines outlined by this committee.

### Sample collection, RNA extraction and qRT-PCR

We selected five vertebrate species, *Manis javanica, Atelerix albiventris, Gallus, and Acipenser schrenckii* and *Shinisaurus crocodilurus* for tissue sampling; among them, *M. javanica* and *S. crocodilurus* individuals died of their wounds. We collected the heart, liver, spleen, lung, kidney, pancreas, brain, testis, and ovary and muscle tissues from male and female adults. A total of 30 samples with an average of five individuals per species were used for RNA extraction using the RNAiso Pure RNA Isolation Kit (Takara, Japan). Total RNA was extracted from 0.25 g of tissue using the manual (TRIzol) method. We designed the primers by using references mRNA sequences retrieved from NCBI Genbank. The Prime Script™RT reagent kit with gDNA eraser was used to remove genomic DNA and to synthesize cDNA. cDNA samples from different tissue samples were assayed by quantitative real-time PCR (qRT-PCR) using specific set of primers (Supplementary Table 7) in the Thermal Cycler Dice® Real Time System (Bio-Rad, Hercules, CA, USA) using TB Green Premix Ex Taq™ II (Perfect Real Time, Cat. # PRO81A/B, Takara Co., Ltd.) with 96-well plates were used and each well contains a reaction mixture of 20μl containing 10μl of TB Green premix, 1.6μl primer mix (0.8μl of each primer) 2μl cDNA and 6.4ul of ddH_2_O. The transcript level was normalized with beta-actin as a housekeeping gene of each representative species.

### Identification, alignment, and filtering of vertebrate immune tolerance genes and orthologs

The orthologs of the PD1, PD-L1, and PD-L2 genes were identified, recovered, aligned, and filtered from the Kyoto Encyclopedia of Genes and Genomes (KEGG) database [55]. These genes were used to identify and retrieve the sequences of human genes from Ensembl BioMart [56]. The gene accession numbers were used to probe coding sequences (CDS) of vertebrate species in the NCBI and Ensembl databases (Supplementary Tables 4–6). Moreover, one-to-one orthologs in the vertebrate species were identified by performing tBLASTn and BLASTn searches [57]. In addition, the homology patterns among the protein-coding genes across the sequenced vertebrate genomes were determined by the OMA v.1.0.0 program [58]. The sequence alignments were made using MAFFT v.7.221 [59]. This aligned set of homologous proteins was used for further analyses.

### Tests of selection

The proportion of sites in positively selected genes across vertebrate lineages was identified by comparison with the nearly neutral model of evolution to identify the signatures of positive selection. Positive selection sites were identified as those with higher nonsynonymous-synonymous substitution ratios (*ω* = *dN/dS*) than expected under neutral evolution, *ω* = 1 [45, 60, 61]. The genes under positive selection with high *ω* values at particular sites across vertebrate phylogenies were identified by using two different tools. Initially, the site models were used [62, 63] and executed in the PAML v4.8 package [61] to compute likelihood values and different constraint estimations for seven evolutionary models. Some genes contained copies; therefore, we performed all selection analyses on the gene trees from all species in the data set and individually for the genes with no duplicates in the species tree. The species tree constructed by OMA was used as the phylogenetic hypothesis [64]. First, we used M0, which evaluates a single *ω* for all positions in the alignment. The branch lengths predicted with M0 were used as fixed branch lengths for subsequent models to reduce computational time. We performed likelihood ratio tests between selection and neutral models (*ω*> 1) to determine the genes under positive selection. The likelihood values from the M1a vs. M2a, M7 vs. M8, and M8 vs. M8a models were compared [62, 63], and the *p*-values were calculated, conferring a χ2 distribution with 2 degrees, 1 degree, and 1 degree of freedom, respectively [65].

Moreover, the genes with signatures of positive selection at a portion of sites were identified by using BUSTED [66], a modelling program executed in the HyPhy package [67]. BUSTED relies on a model that permits branch-to-branch variations across the whole tree [66]. Furthermore, the signals of positive selection for immune-tolerant genes were redetected by estimating the rates of nonsynonymous to synonymous substitutions at individual sites in the aligned sequences using various likelihood models, including the fixed effect likelihood (FEL), internal fixed effect likelihood (IFEL), single likelihood ancestor counting (SLAC), and maximum-likelihood estimation (MEME) methods [67–69]. To further confirm codon sites under selection pressure, aligned sequences of selected genes were tested in Selecton version 2.2 [41] (http://selecton.tau.ac.il/). Selecton allows the *ω* ratio to shift among different codons within the multiple sequence alignment, and this was estimated by the maximum-likelihood value via the Bayesian inference method [60, 70]. Moreover, the results from Selecton were visualized with color scales that indicated different types of selection.

### Gene enrichment and conserved synteny analyses

The conserved synteny patterns of the PD1, PD-L1, and PD-L2 genes were determined using the Genomicus v.91.01 [71] and Ensembl [72] databases. We evaluated the conserved synteny for the genomic regions neighboring the PD1, PD-L1 and PD-L2 genes in vertebrate species. The evolutionary novelty of these genes was analyzed by using the Protein Historian program to recognize the taxon of sources of these genes [73]. Conserved syntenies might be linked with gene function and the corresponding gene expression [74, 75]; therefore, we used enrichment analysis to evaluated the biological significance of syntenic genes by searching these genes in various programs: EnrichNet, a network-based enrichment analysis [76], and ConsensusPathDB, a meta-database [77]. EnrichNet detects the genes in specific molecular systems and, using an arbitrary selection, estimates the intervals between the genes and pathways in a reference catalog [76]. ConsensusPathDB is a meta-database that contains an extensive assembly of human molecular interaction data linked to various public sources and has been used for reporting interaction network units and enrichment analyses [78]. The functional motifs and domains of the PD1, PD-L1 and PD-L2 proteins were predicted using the MEME tool (http://meme-suite.org/).

### Three-dimensional (3D) protein modeling and structural analysis

The crystal structures of human PD1, PD-L1 and PD-L2 were generated using the Swiss model (https://swissmodel.expasy.org) online tool [79] and Phyre2 (http://www.sbg.bio.ic.ac.uk/phyre2/html). The homology modeling method was used to predict protein structure. The 3D structures of PD1, PD-L1, PD-L2 were predicted by the I-TESSAR and Swiss modeling approaches [80]. The assembled target proteins were minimized by using the Amber force field and the conjugate gradient algorithm in UCSF Chimera 1.10.1 [81]. Furthermore, the ProSA web server [82] was used to evaluate the stereochemical characteristics of the predicted structures.

### Conservation analysis

The ConSurf server (consurf.tau.ac.il/) was used to evaluate the evolutionary conservation of amino acid residues of the human PD1, PD-L1 and PD-L2 proteins [83]. The amino acids are more conserved and are essential for protein interactions or are present within more enzymatic pockets than other amino acids of the protein. Therefore, the changes in the conserved amino acids are more lethal than polymorphisms located in flexible regions in a protein because they disrupt the protein function and structure [84, 85]. The conserved amino acids were predicted based on conservation values ranging from 1 to 9; a conservation value between 1–4 is considered variable, a value of 5–6 exhibits average conservation and a value ranging from 7 to 9 indicates very high conservation [86].

### Bioinformatics analysis of protein sequences

The structures of the PD1, PD-L1 and PD-L2 proteins, including protein-binding positions, solvent accessibility, and disordered structures, were predicted using the Predict Protein server [87]. The secondary structures of the PD1, PD-L1, and PD-L2 proteins were predicted by the CFSSP program [54, 88]. Hydrophobic cluster analysis (HCA) was performed using the HCA 1.0.2 program [89] via the Mobyle@RPBS web portal and framework [90].

### Coevolution analysis of protein residues

We used a web-based tool (CoeViz) [91] that allows the focused and rapid analysis of protein topographies, such as functional sites and structural domains, and that delivers a variable analysis and visualization of pairwise coevolution of amino acid residues. Full protein sequences of PD1, PD-L1 and PD-L2 were obtained from CoeViz analysis via χ2 covariance metrics [92] and were adjusted for phylogenetic bias in the MSA to predict the maps of covarying residues and the large overlaps with functional regions and the known domains of the protein. The visualization of the residue interactions was improved with circular drawings, and the residues were highlighted in the protein sequences and 3D structures.

## Supplementary Material

Supplementary Figures

Supplementary Tables
