# Supplementary material for: Adaptive selection in the evolution of programmed cell death-1 and its ligands in vertebrates
Source: Aging (Albany NY). 2020 Feb 11;12(4):3516–57. doi: 10.18632/aging.102827 (PMC7066927; doi:10.18632/aging.102827)
Supplement: Supplementary Figures [file aging-12-102827-s002..pdf]

SUPPLEMENTARY FIGURES

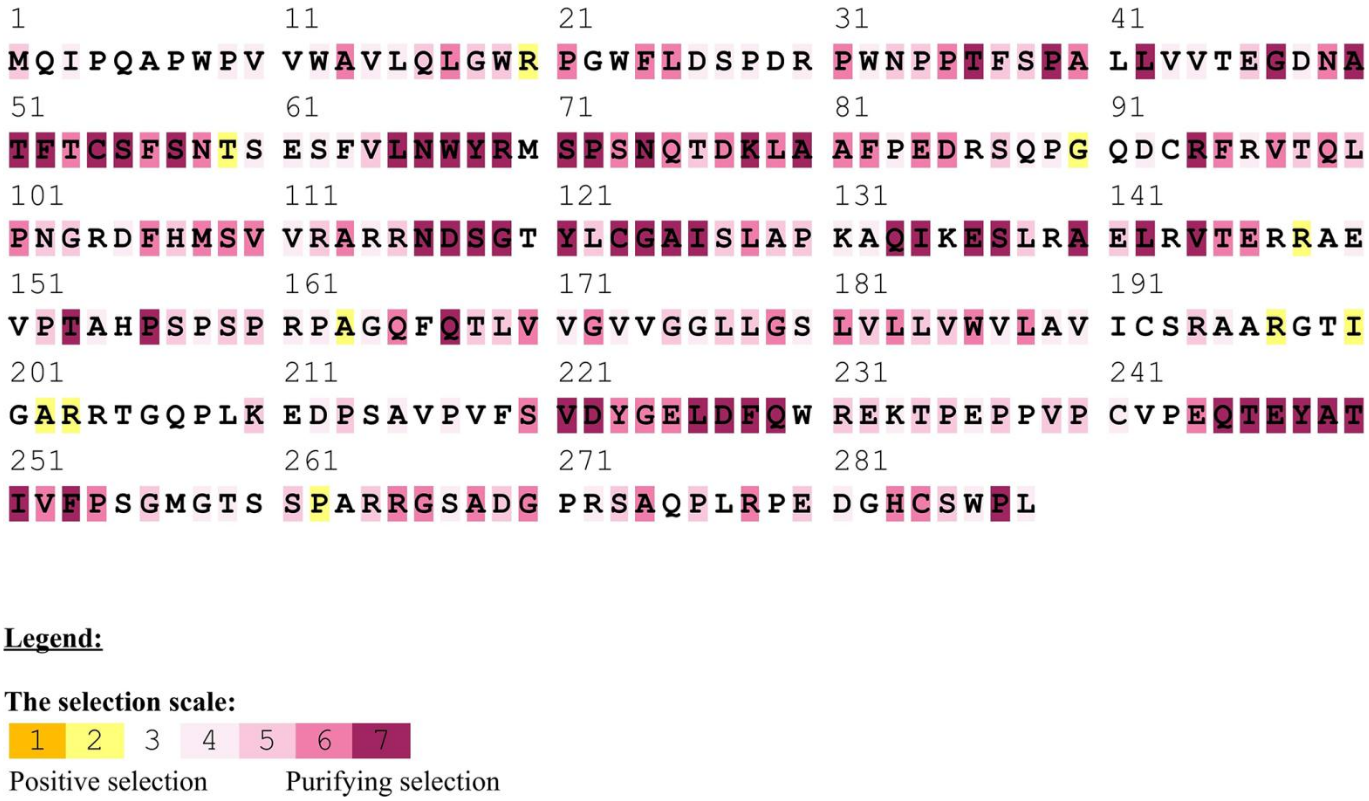

Supplementary Figure 1. Detection of positive/purifying selection in PD1 homologous sequences from vertebrates. Color-coded results of Selecton analyses of human PD1, compared to sequences from 52 aligned nucleotide coding sequences. Selection pressure was measured on sequences using mechanistic-empirical combination (MEC) model of Selecton version 2.2. Yellow and brown highlights represent positive selection, grey and white highlights represent a neutral selection and purple highlight represent purifying selection on codons.

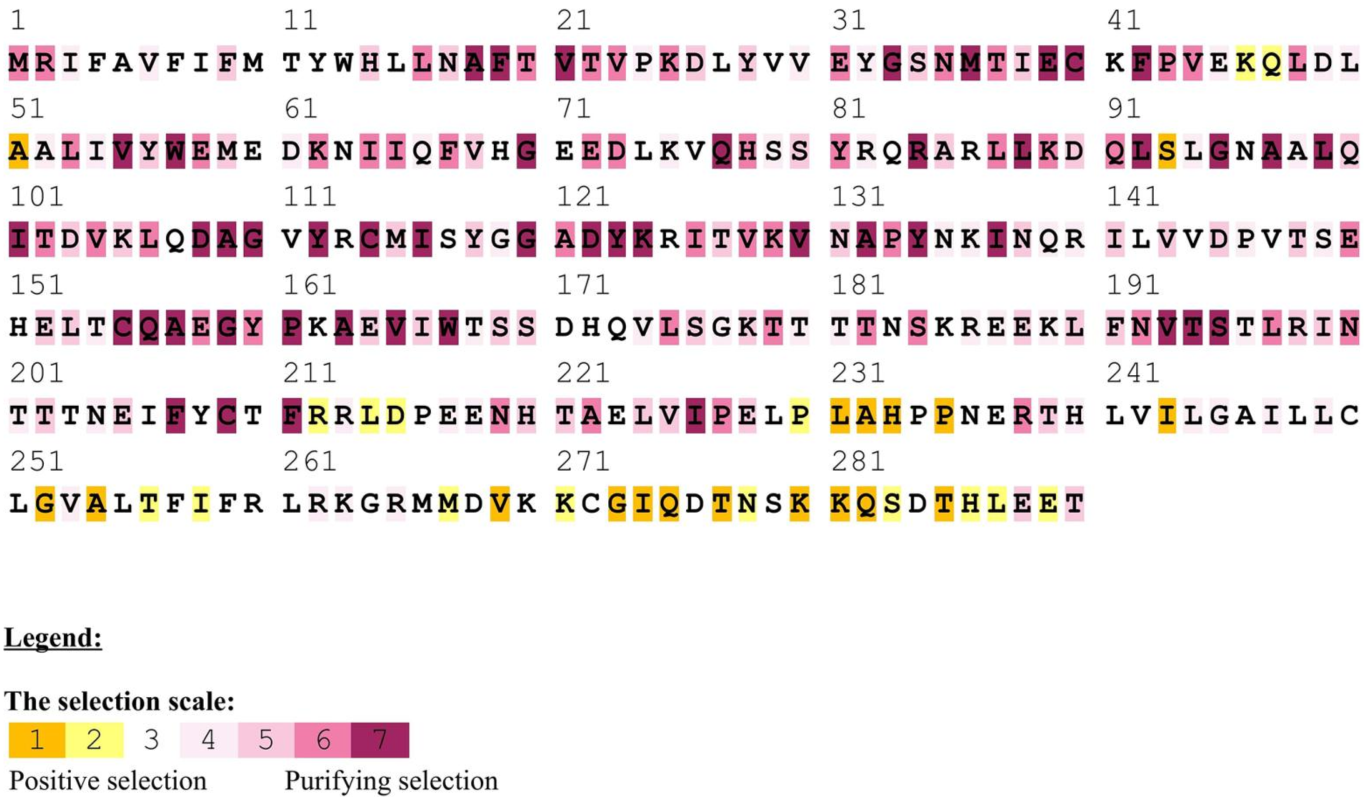

Supplementary Figure 2. Detection of positive/purifying selection in PD-L1 homologous sequences from vertebrates. Color-coded results of Selecton analyses of human PD-L1, compared to sequences from 58 aligned nucleotide coding sequences. Selection pressure was measured on sequences using mechanistic-empirical combination (MEC) model of Selecton version 2.2. Yellow and brown highlights represent positive selection, grey and white highlights represent a neutral selection and purple highlight represent purifying selection on codons.

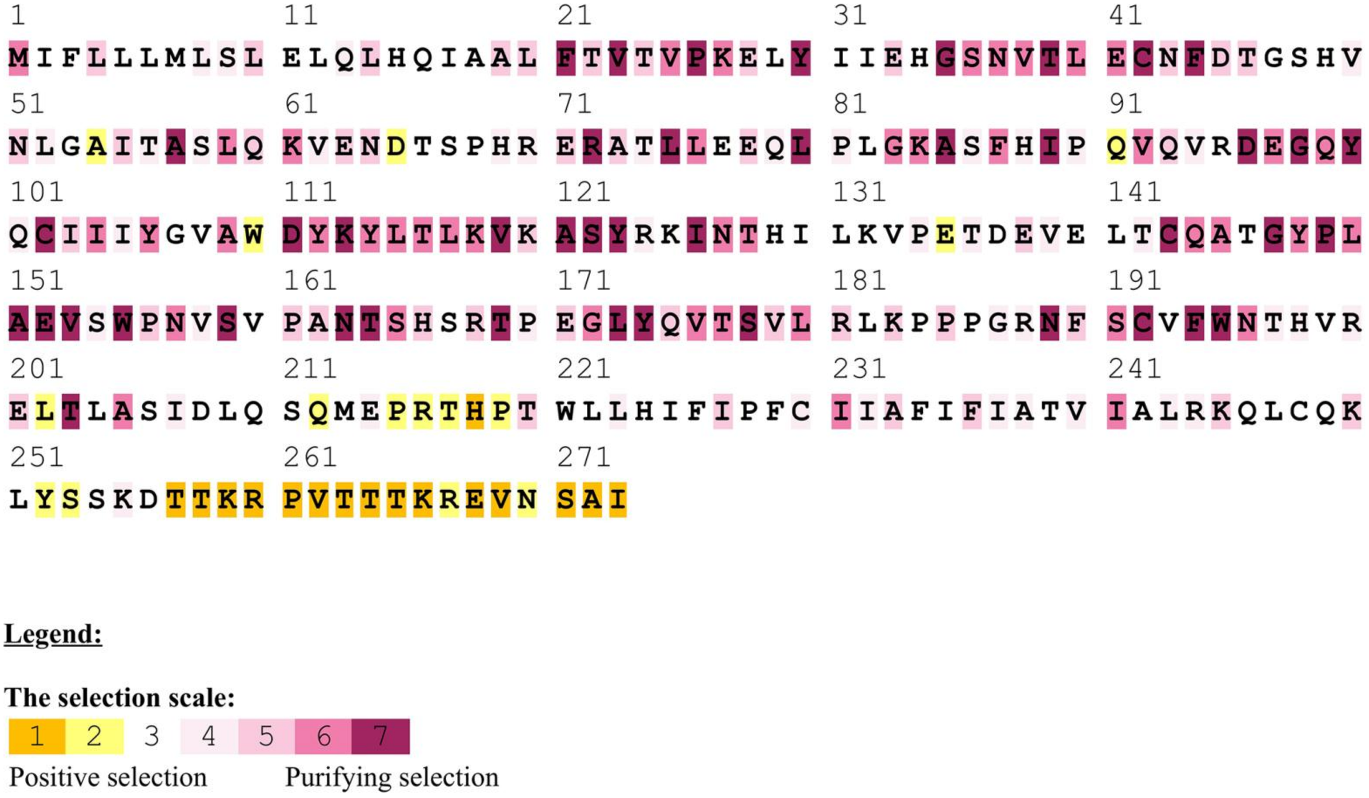

Supplementary Figure 3. Detection of positive/purifying selection in PD-L2 homologous sequences from vertebrates. Color-coded results of Selecton analyses of human PD-L2, compared to sequences from 56 aligned nucleotide coding sequences. Selection pressure was measured on sequences using mechanistic-empirical combination (MEC) model of Selecton version 2.2. Yellow and brown highlights represent positive selection, grey and white highlights represent a neutral selection and purple highlight represent purifying selection on codons.

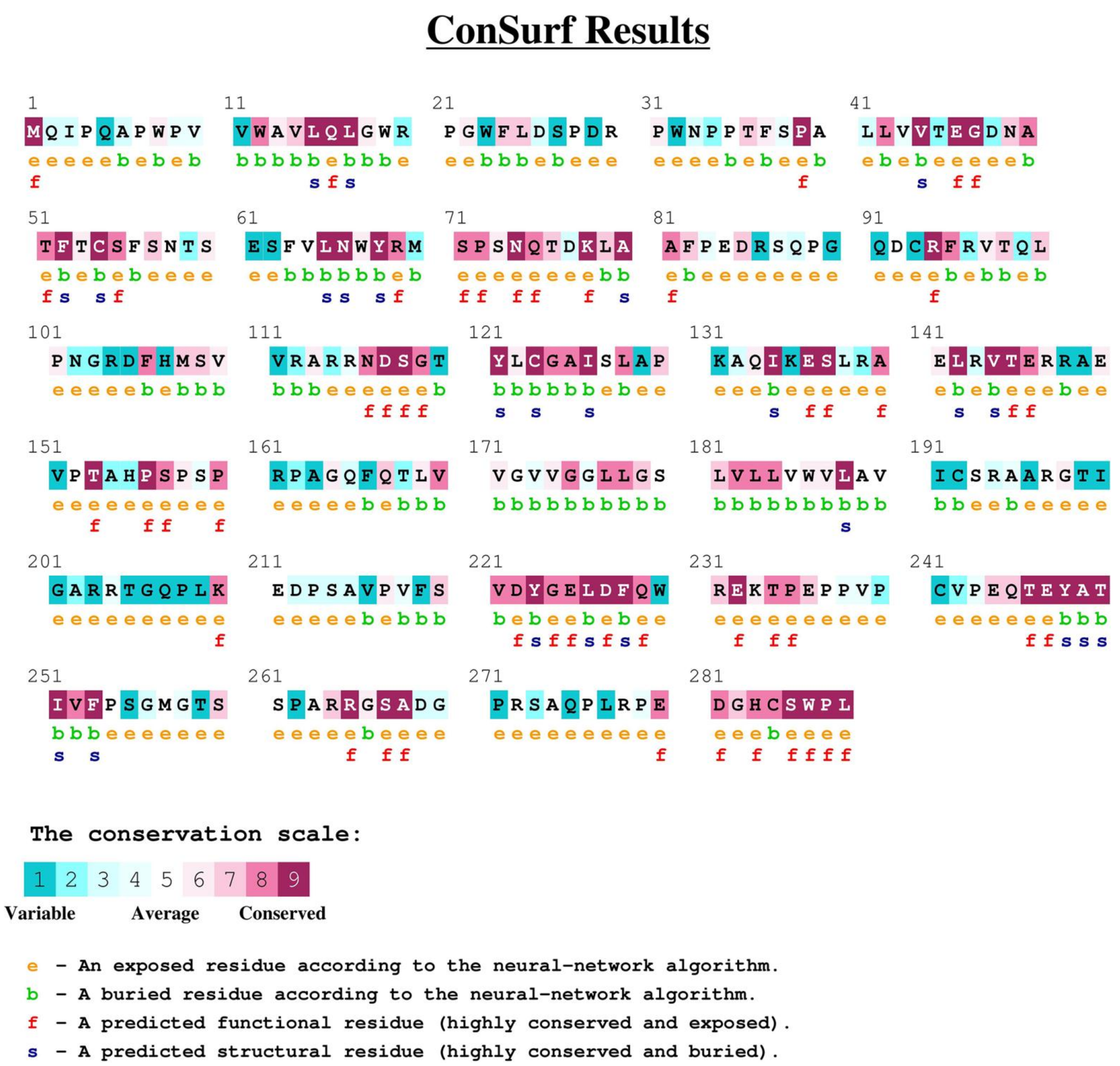

Supplementary Figure 4. ConSurf output of PD1, using the UniRef90 protein database. Colors of the ConSurf output indicate the level of sequence conservation. Purple indicates conservation and blue indicates variability. Residues are predicted to be exposed (e), buried (b), highly conserved and exposed (f), or highly conserved and buried, (s).

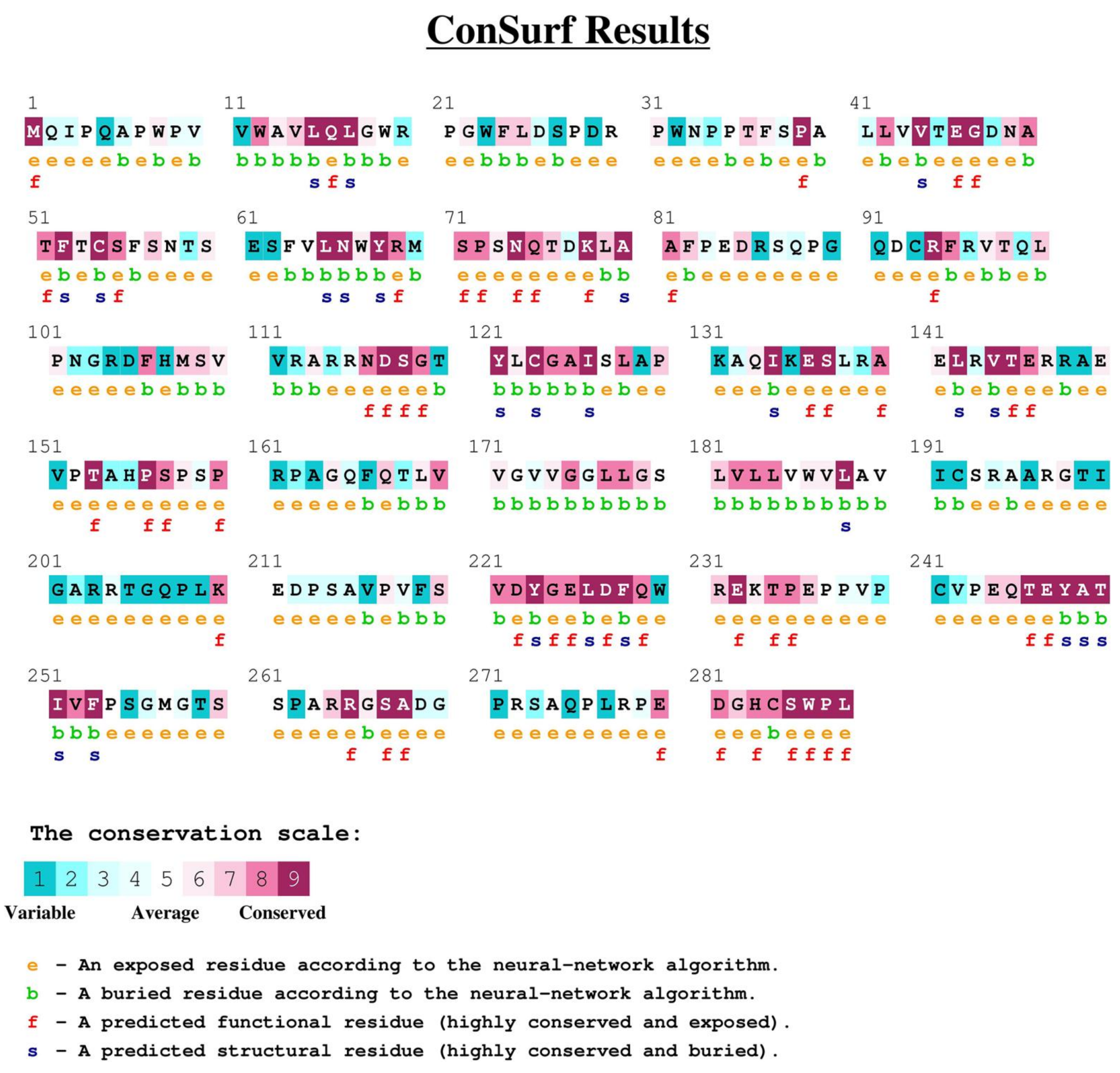

Supplementary Figure 5. ConSurf output of PD-L1, using the UniRef90 protein database. Colors of the ConSurf output indicate the level of sequence conservation. Purple indicates conservation and blue indicates variability. Residues are predicted to be exposed (e), buried (b), highly conserved and exposed (f), or highly conserved and buried, (s).

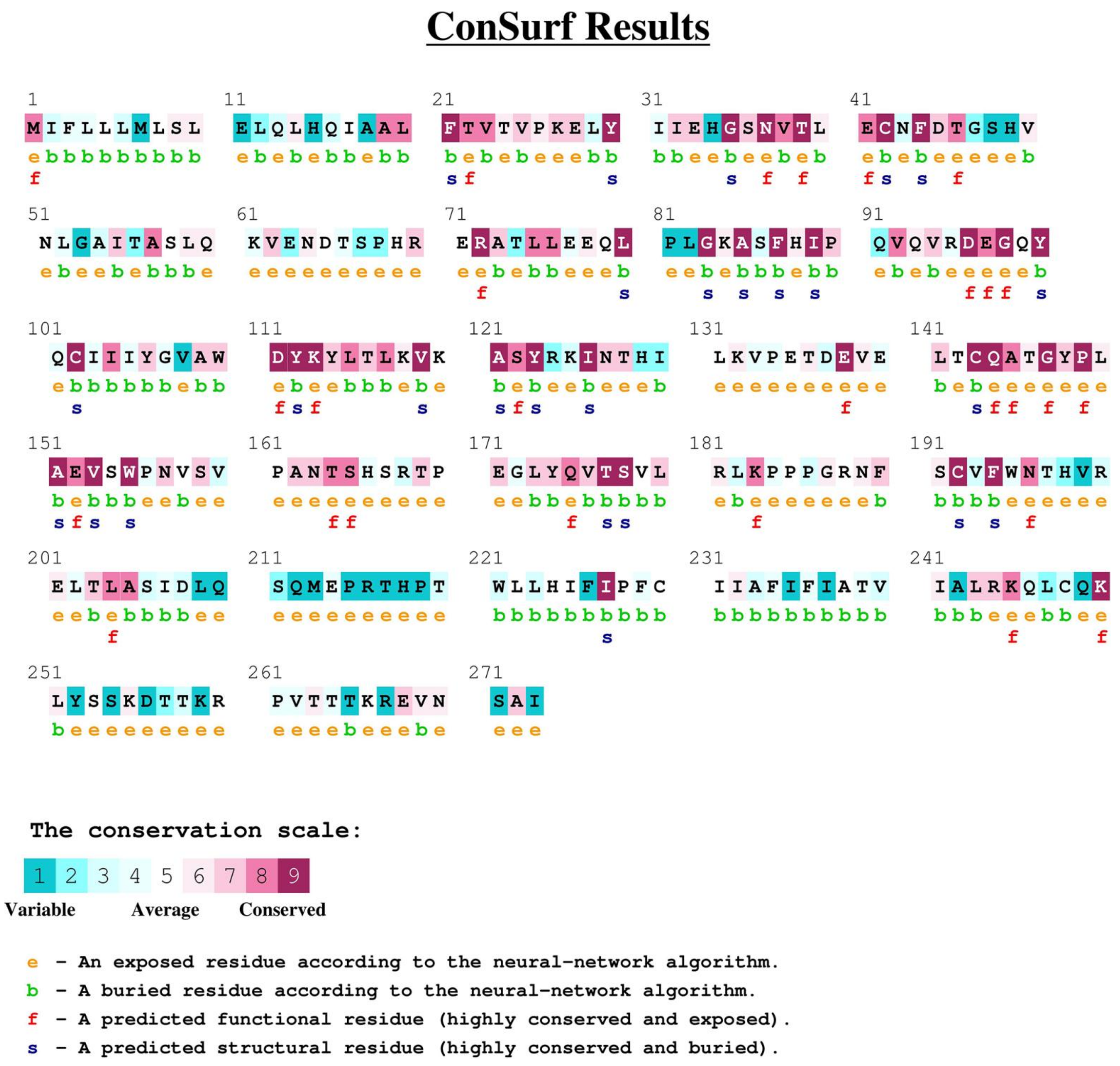

Supplementary Figure 6. ConSurf output of PD-L2, using the UniRef90 protein database. Colors of the ConSurf output indicate the level of sequence conservation. Purple indicates conservation and blue indicates variability. Residues are predicted to be exposed (e), buried (b), highly conserved and exposed (f), or highly conserved and buried, (s).

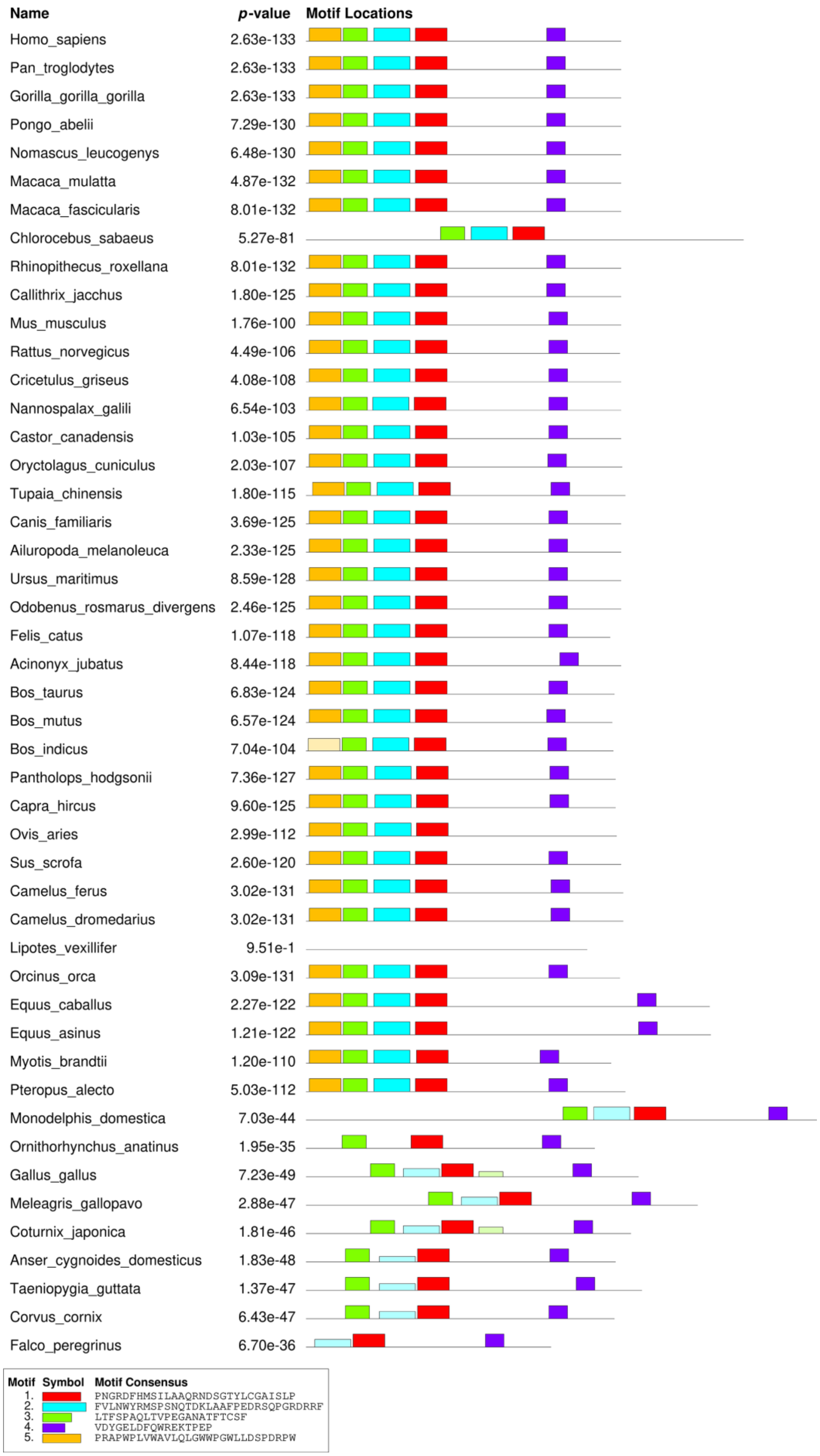

Supplementary Figure 7. Motif distribution of PD1 gene in the vertebrate species. Motifs of PD1 gene from 52 species are predicted using MEME suite (http://meme.nbcr.net/meme/) based on amino acids sequences. All sequences are separated by 5 conservative motifs with colors, including motif 1 (red), motif 2 (cyan), motif 3 (green), motif 4 (purple) and motif 5 (brown).

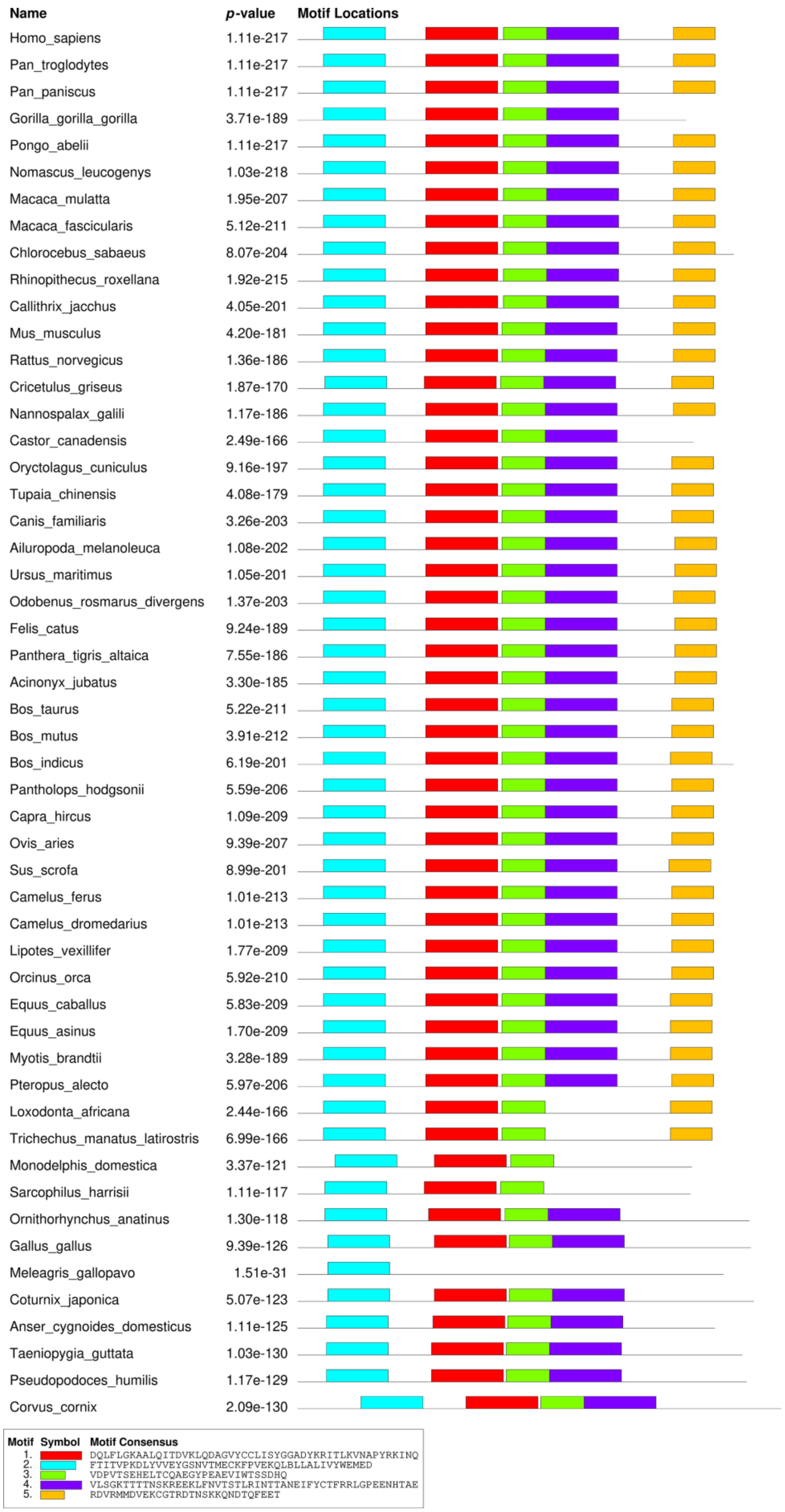

Supplementary Figure 8. Motif distribution of PD-L1 gene in the vertebrate species. Motifs of PD-L1 gene from 58 species are predicted using MEME suite (http://meme.nbcr.net/meme/) based on amino acids sequences. All sequences are separated by 5 conservative motifs with colors, including motif 1 (red), motif 2 (cyan), motif 3 (green), motif 4 (purple) and motif 5 (brown).

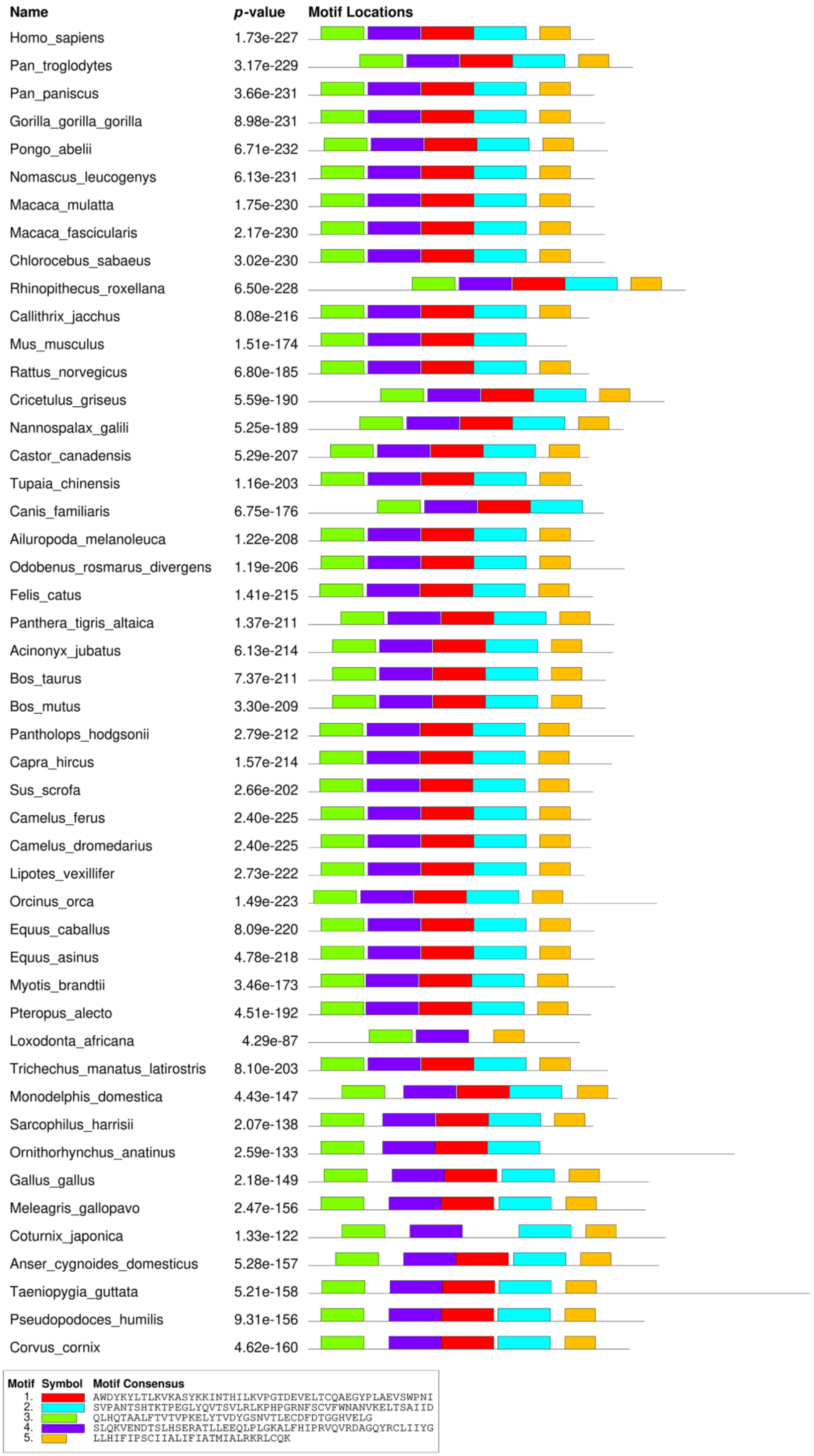

Supplementary Figure 9. Motif distribution of PD-L2 gene in the vertebrate species. Motifs of PD-L2 gene from 56 species are predicted using MEME suite (http://meme.nbcr.net/meme/) based on amino acids sequences. All sequences are separated by 5 conservative motifs with colors, including motif 1 (red), motif 2 (cyan), motif 3 (green), motif 4 (purple) and motif 5 (brown).

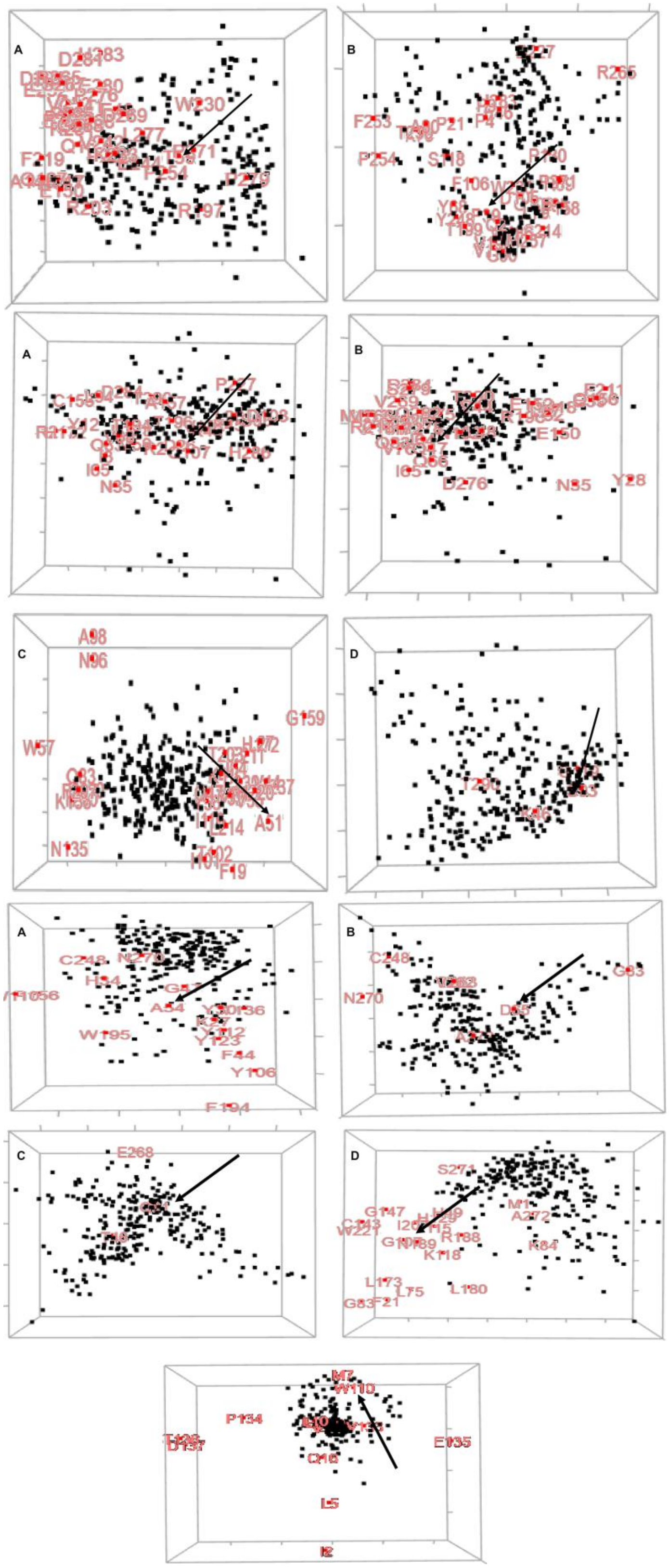

Supplementary Figure 10. 3D multidimensional scaling (MDS) scatterplots of co-varying residues in human PD1, PD-L1, and PD-L2. (**A**) Highlighted red are the residues corresponding to the positively selected residue. Both black and red dots can be optionally labeled with the residue identifiers.

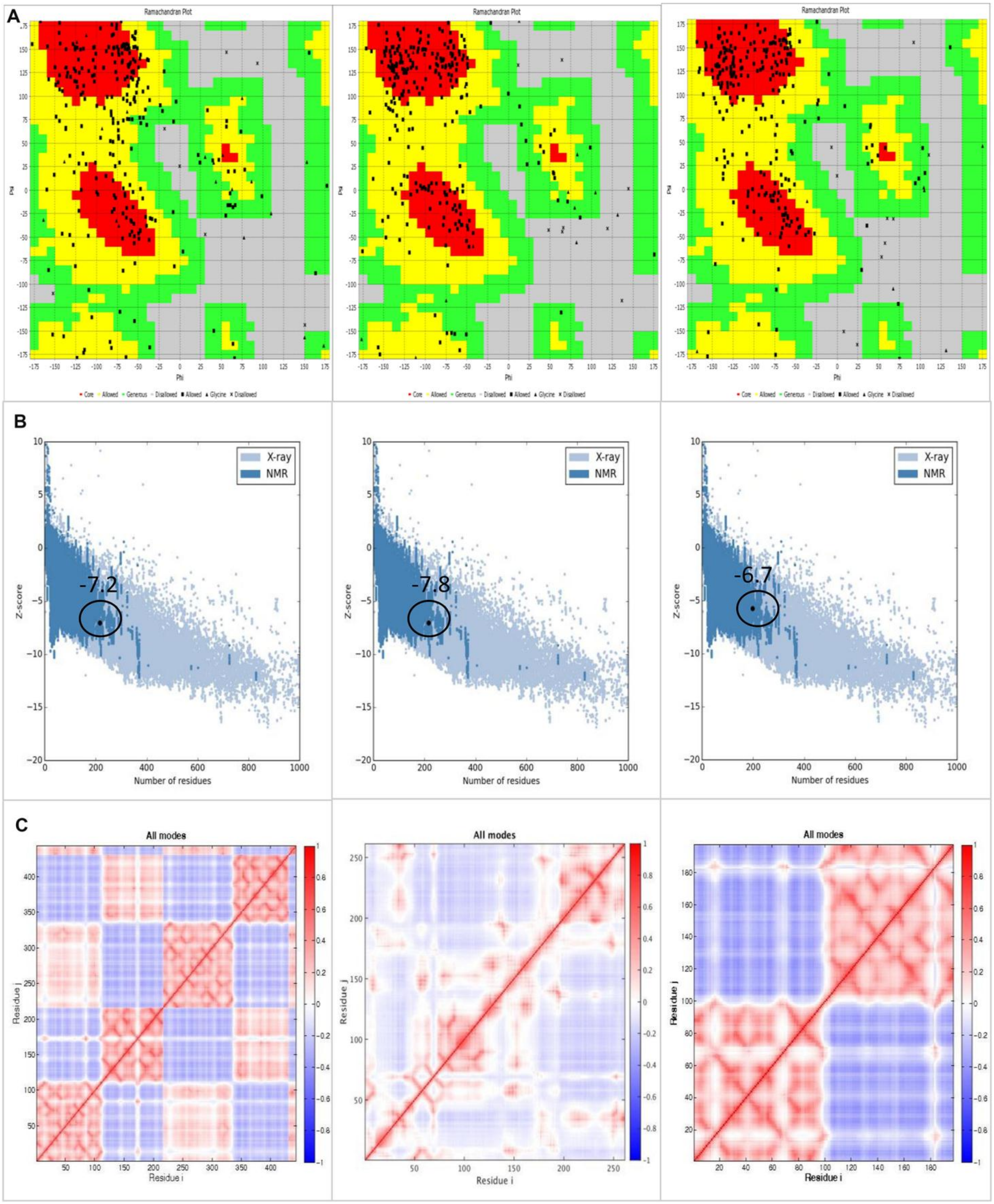

Supplementary Figure 11. (**A**) Ramachandran plot analysis of PD1, PDL1 and PDL2 proteins. The backbone conformation angles for respective residue in the modeled protein crystal structures. Red color indicates the core region, yellow indicates allowed region, green is allowed region, and grey is disallowed region. (**B**) Z-score displays the quality analysis of predicted structures. (**C**) The cross-correlation analysis of all predicted structures. Positive correlations are plotted in the upper left triangle and negative correlations in the lower right triangle. Open squares: functional hydrophobic and negative residues.
