# Supplementary material for: Adaptive selection in the evolution of programmed cell death-1 and its ligands in vertebrates
Source: Aging (Albany NY). 2020 Feb 11;12(4):3516–57. doi: 10.18632/aging.102827 (PMC7066927; doi:10.18632/aging.102827)
Supplement: Supplementary Tables [file aging-12-102827-s001..pdf]

SUPPLEMENTARY TABLES

Supplementary Table 1. An adaptive branch-site (aBSREL) test for episodic diversification of the PD1 gene.

Name	B	LRT	Test p-value	Uncorrected p-value	ω distribution over sites
CHLOROCEBUS_SABAEUS	0.221	151.8573	0	0	ω1 = 0.0287 (72%)<br>ω2 = 197 (28%)
ACINONYX_JUBATUS	0.1127	134.3588	0	0	ω1 = 0.0326 (84%)<br>ω2 = 229 (16%)
OVIS_ARIES	0.1765	148.3166	0	0	ω1 = 0.732 (79%)<br>ω2 = ∞ (21%)
TAENIOPYGIA_GUTTATA	0.0778	57.4098	0	0	ω1 = 0.361 (93%)<br>ω2 = 745 (6.9%)
CHRYSEMYS_PICTA	0.1014	84.2906	0	0	ω1 = 0.205 (84%)<br>ω2 = ∞ (16%)
Node47	0.0143	37.3204	0	0	ω1 = 0.00 (92%)<br>ω2 = 131 (7.8%)
Node38	0.1042	25.0463	0.0001	0	ω1 = 0.441 (89%)<br>ω2 = 5000 (11%)
ALLIGATOR_SINENSIS	0.2851	22.3465	0.0004	0	ω1 = 0.288 (72%)<br>ω2 = 20.3 (28%)
Node37	0.1629	18.77	0.0025	0	ω1 = 0.114 (72%)<br>ω2 = 56.3 (28%)
CORVUS_CORNIX	0.0603	17.9611	0.0037	0	ω1 = 0.109 (95%)<br>ω2 = 35.9 (4.6%)
Node36	0.0821	15.1483	0.0151	0.0002	ω1 = 0.00 (74%)<br>ω2 = 93.5 (26%)
APTERYX_AUSTRALIS_MANTELLI	0.1045	14.5227	0.0205	0.0002	ω1 = 0.244 (95%)<br>ω2 = ∞ (5.5%)

B; Optimized branch length
LRT: Likelihood ratio test statistic for ω+ = 1 (null) versus ω+ unrestricted (alternative)
Test p-value: The p-value corrected for multiple testing.
Uncorrected P-value: The uncorrected p-value for the LRT test.

Supplementary Table 2. An adaptive branch-site (aBSREL) test for episodic diversification of PD-L1 gene.

Name	B	LRT	Test p-value	Uncorrected p-value	ω distribution over sites
COTURNIX_JAPONICA	0.0481	28.7232	0	0	ω1 = 0.298 (96%)<br>ω2 = 5710 (3.7%)
TAENIOPYGIA_GUTTATA	0.0734	33.334	0	0	ω1 = 0.190 (94%)<br>ω2 = 283 (5.8%)
PSEUDOPODOCES_HUMILIS	0.0672	41.2926	0	0	ω1 = 0.474 (93%)<br>ω2 = 90.4 (6.8%)
FALCO_PEREGRINUS	0.1009	63.2226	0	0	ω1 = 0.691 (89%)<br>ω2 = 212 (11%)
APTERYX_AUSTRALIS_MANTELLI	0.0976	28.7185	0	0	ω1 = 0.376 (92%)<br>ω2 = 187 (8.5%)
ANSER_CYGNOIDES_DOMESTICUS	0.1042	22.369	0.0005	0	ω1 = 0.340 (95%)<br>ω2 = 5710 (5.3%)
Node41	0.0217	19.3186	0.0022	0	ω1 = 0.00 (92%)<br>ω2 = 5710 (8.4%)
CHLOROCEBUS_SABAEUS	0.0247	16.2851	0.0102	0.0001	ω1 = 0.385 (98%)<br>ω2 = 1660 (1.6%)
Node33	0.1354	15.022	0.0191	0.0002	ω1 = 0.295 (88%)<br>ω2 = 57.4 (12%)
SARCOPHILUS_HARRISII	0.1369	13.2103	0.0471	0.0005	ω1 = 0.216 (94%)<br>ω2 = 30.1 (5.7%)

B; Optimized branch length
LRT: Likelihood ratio test statistic for ω+ = 1 (null) versus ω+ unrestricted (alternative)
Test p-value: The p-value corrected for multiple testing.
Uncorrected P-value: The uncorrected p-value for the LRT test.

Supplementary Table 3. An adaptive branch-site (aBSREL) test for episodic diversification of PD-L2 gene.

Name	B	LRT	Test p-value	Uncorrected p-value	ω distribution over sites
CRICETULUS_GRISEUS	0.1001	40.3338	0	0	ω1 = 0.359 (88%)<br>ω2 = 211 (12%)
PANTHOLOPS_HODGSONII	0.0216	56.1676	0	0	ω1 = 0.00 (92%)<br>ω2 = 1650 (7.7%)
LOXODONTA_AFRICANA	0.2699	71.5397	0	0	ω1 = 0.831 (71%)<br>ω2 = ∞ (29%)
TAENIOPYGIA_GUTTATA	0.0998	147.3444	0	0	ω1 = 0.437 (81%)<br>ω2 = 5000 (19%)
CORVUS_CORNIX	0.0583	47.1431	0	0	ω1 = 0.565 (94%)<br>ω2 = 204 (6.3%)
Node68	0.0732	37.2754	0	0	ω1 = 0.308 (96%)<br>ω2 = 5000 (3.9%)
Node84	0.0156	43.9788	0	0	ω1 = 0.421 (89%)<br>ω2 = 1940 (11%)
PANTHERA_TIGRIS_ALTAICA	0.016	24.5613	0.0001	0	ω1 = 0.762 (97%)<br>ω2 = 11400 (2.7%)
Node23	0.03	24.5946	0.0001	0	ω1 = 0.133 (89%)

					ω2 = 5000 (11%)
Node41	0.0311	24.1356	0.0002	0	ω1 = 1.00 (89%)<br>ω2 = ∞ (11%)
CHRYSEMYS_PICTA	0.1462	21.302	0.0007	0	ω1 = 0.457 (91%)<br>ω2 = 40.8 (9.4%)
NANNOSPALAX_GALILI	0.1469	20.5087	0.0011	0	ω1 = 0.359 (91%)<br>ω2 = ∞ (9.3%)
LIPOTES_VEXILLIFER	0.0136	20.2389	0.0013	0	ω1 = 0.450 (98%)<br>ω2 = 1530 (1.6%)
Node55	0.0103	20.1447	0.0013	0	ω1 = 1.00 (97%)<br>ω2 = 90200 (2.6%)
COTURNIX_JAPONICA	0.1582	19.8778	0.0015	0	ω1 = 0.602 (91%)<br>ω2 = 118 (8.7%)
ACINONYX_JUBATUS	0.0158	19.1886	0.0021	0	ω1 = 0.891 (99%)<br>ω2 = 5820 (0.69%)
Node75	0.1565	16.6122	0.0074	0	ω1 = 0.278 (87%)<br>ω2 = 2180 (13%)
Node5	0.0019	15.603	0.0122	0	ω1 = 0.0169 (95%)<br>ω2 = ∞ (5.1%)
Node82	0.09	15.1548	0.0151	0	ω1 = 0.706 (83%)<br>ω2 = 289 (17%)
Node14	0.0014	15.0154	0.016	0	ω1 = 0.0638 (96%)<br>ω2 = ∞ (3.8%)
Node38	0.008	14.2666	0.023	0	ω1 = 0.00 (93%)<br>ω2 = ∞ (7.0%)
CALLITHRIX_JACCHUS	0.0396	14.0674	0.0252	0	ω1 = 0.331 (97%)<br>ω2 = 158 (2.7%)
ORNITHORHYNCHUS_ANATINUS	0.3646	13.9868	0.0259	0	ω1 = 0.114 (74%)<br>ω2 = ∞ (26%)

B; Optimized branch length
LRT: Likelihood ratio test statistic for ω+ = 1 (null) versus ω+ unrestricted (alternative)
Test p-value: The p-value corrected for multiple testing.
Uncorrected P-value: The uncorrected p-value for the LRT test

Supplementary Table 4. The detail information of PD1 gene in vertebrate species.

Species	Scientific Name	NCBI-Protein ID	Ensembl	Uniprot
Human	*Homo sapiens*	NP_005009	ENSG00000188389	A0A0M3M0G7
Chimpanzee	*Pan troglodytes*	XP_016806440		A0A2I3SUZ1
Western lowland gorilla	*Gorilla gorilla gorilla*	XP_004033550	ENSGGOG00000014312	G3REH1
Sumatran orangutan	*Pongo abelii*	XP_024099392	ENSPPYG00000013360	
Northern white-cheeked gibbon	*Nomascus leucogenys*	XP_003282018		G1R8C2
Rhesus monkey	*Macaca mulatta*	NP_001107830	ENSMMUG00000008592	B0LAJ2
Crab-eating macaque	*Macaca fascicularis*	NP_001271065		B0LAJ3
Green monkey	*Chlorocebus sabaeus*	XP_007965216	ENSCSAG00000005736	
Golden snub-nosed monkey	*Rhinopithecus roxellana*	XP_010375274		A0A2K6QL35
White-tufted-ear marmoset	*Callithrix jacchus*	XP_002750041	ENSCJAG00000019795	
Mouse	*Mus musculus*	NP_032824	ENSMUSG00000026285	Q544F3
Rat	*Rattus norvegicus*	NP_001100397	ENSRNOG00000049797	D3ZIN8
Chinese hamster	*Cricetulus griseus*	XP_003499314		
Mole Rat	*Nannospalax galili*	XP_008827391		
American beaver	*Castor canadensis*	XP_020013289		
Rabbit	*Oryctolagus cuniculus*	XP_017195300	ENSOCUG00000008079	G1SUF0
Chinese tree shrew	*Tupaia chinensis*	XP_006165239		
Dog	*Canis familiaris*	NP_001301026	ENSCAFG00000013184	A0A090BAM7
Giant panda	*Ailuropoda melanoleuca*	XP_002923181	ENSAMEG00000016373	G1MD70
Polar bear	*Ursus maritimus*	XP_008688483		A0A384C0X7
Pacific walrus	*Odobenus rosmarus divergens*	XP_004412778		A0A2U3WTR3
Domestic cat	*Felis catus*	NP_001138982		A9YUA6
Amur tiger	*Panthera tigris altaica*	XP_007094211		
Cheetah	*Acinonyx jubatus*	XP_026912119		
Cow	*Bos taurus*	NP_001076975	ENSBTAG00000011543	A4FV85
Wild yak	*Bos mutus*	XP_005901569		
Chiru	*Pantholops hodgsonii*	XP_005959703		
Goat	*Capra hircus*	XP_013818552		
Sheep	*Ovis aries*	XP_012031617	ENSOARG00000017587	
Pig	*Sus scrofa*	NP_001191308		
Wild Bactrian camel	*Camelus ferus*	XP_014412330		
Arabian camel	*Camelus dromedarius*	XP_010986792		
killer whale	*Orcinus orca*	XP_004262561		
Horse	*Equus caballus*	XP_023498581		
Ass	*Equus asinus*	XP_014718129		
Brandt's bat	*Myotis brandtii*	XP_005857730		
Black flying fox	*Pteropus alecto*	XP_006909981		
Opossum	*Monodelphis domestica*	XP_016280819	ENSMODG00000029268	
Platypus	*Ornithorhynchus anatinus*	XP_007667778		
Chicken	*Gallus gallus*	XP_422723	ENSGALG00000046559	
Turkey	*Meleagris gallopavo*	XP_010715066		
Japanese quail	*Coturnix japonica*	XP_015727433		
Swan goose	*Anser cygnoides domesticus*	XP_013050415		
Zebra finch	*Taeniopygia guttata*	XP_012431056		
Hooded crow	*Corvus cornix*	XP_019140093		

Peregrine falcon	*Falco peregrinus*	XP_013154882	
Rock pigeon	*Columba livia*	XP_005513327	A0A2I0LU23
Brown kiwi	*Apteryx australis mantelli*	XP_013814564	
Western clawed frog	*Xenopus tropicalis*	XP_004914438	
Chinese alligator	*Alligator sinensis*	XP_025058540	
Western painted turtle	*Chrysemys picta*	XP_005311833	
Green sea turtle	*Chelonia mydas*	XP_007057904	

List of species and NCBI GenBank accession numbers for sequences used to construct the datasets for hypothesis testing.

Supplementary Table 5. The detail information of PD-L1 gene in vertebrate species.

Species	*Scientific Name*	Ensembl	Uniprot	NCBI-Protein ID
Human	*Homo sapiens*	ENSG00000120217	Q9NZQ7	
Chimpanzee	*Pan troglodytes*	ENSPTRG00000020755	H2QWZ8	
Bonobo	*Pan paniscus*		A0A2R9B063	XP_003823426
Western lowland gorilla	*Gorilla gorilla gorilla*			XP_018889139
Sumatran orangutan	*Pongo abelii*	ENSPPYG00000019237	H2PS75	XP_002819859
Northern white-cheeked gibbon	*Nomascus leucogenys*		G1RCP1	XP_003273874
Rhesus monkey	*Macaca mulatta*	ENSMMUG00000012235	A4GW29	NP_001077358
Crab-eating macaque	*Macaca fascicularis*		G7PSE7	XP_005581836
Green monkey	*Chlorocebus sabaeus*	ENSCSAG00000008879		XP_007967522
Golden snub-nosed monkey	*Rhinopithecus roxellana*		A0A2K6PJ04	XP_010363385
White-tufted-ear marmoset	*Callithrix jacchus*	ENSCJAG00000011388	A4GW22	NP_001254676
Mouse	*Mus musculus*	ENSMUSG00000016496	Q9EP73	NP_068693
Rat	*Rattus norvegicus*	ENSRNOG00000016112	D4AE25	NP_001178883
Chinese hamster	*Cricetulus griseus*			XP_007626098
Mole Rat	*Nannospalax galili*			XP_008849411
American beaver	*Castor canadensis*			XP_020015185
Rabbit	*Oryctolagus cuniculus*	ENSOCUG00000008117	G1SUI3	XP_008253343
Chinese tree shrew	*Tupaia chinensis*			XP_006152480
Dog	*Canis familiaris*	ENSCAFG00000002120	E2RKZ5	NP_001278901
Giant panda	*Ailuropoda melanoleuca*	ENSAMEG00000009800		XP_011228696
Polar bear	*Ursus maritimus*		A0A384C2Q2	XP_008689127
Pacific walrus	*Odobenus rosmarus divergens*		A0A2U3ZNI8	XP_012420554
Domestic cat	*Felis catus*	ENSFCAG00000009047	M3WAP8	XP_006939101
Amur tiger	*Panthera tigris altaica*			XP_007094214
Pangolin	*Manis javanica*			XM_017657766.1
Cheetah	*Acinonyx jubatus*			XP_014919968
Cow	*Bos taurus*	ENSBTAG00000000095	C5NU11	NP_001156884
Wild yak	*Bos mutus*		L8IU19	XP_005891421
Zebu cattle	*Bos indicus*			XP_019821547
Chiru	*Pantholops hodgsonii*			XP_005983887
Goat	*Capra hircus*			XP_005683750
Sheep	*Ovis aries*	ENSOARG00000013509		XP_004004411
Pig	*Sus scrofa*	ENSSSCG00000005211	Q4QTK1	NP_001020392
Wild Bactrian camel	*Camelus ferus*			XP_014416016

Arabian camel	*Camelus dromedarius*			XP_010991731
Yangtze River dolphin	*Lipotes vexillifer*		A0A340X5J4	XP_007454584
killer whale	*Orcinus orca*			XP_004279158
Horse	*Equus caballus*	ENSECAG00000016312	F7DZ76	XP_001492892
Ass	*Equus asinus*			XP_014716218
Brandt's bat	*Myotis brandtii*			XP_005861392
Black flying fox	*Pteropus alecto*		L5K6N3	XP_006918439
African savanna elephant	*Loxodonta africana*			XP_010586356
Florida manatee	*Trichechus manatus latirostris*		A0A2Y9DG34	XP_004373536
Opossum	*Monodelphis domestica*	ENSMODG00000015352		XP_007499604
Tasmanian devil	*Sarcophilus harrisii*	ENSSHAG00000002682		XP_012399523
Platypus	*Ornithorhynchus anatinus*	ENSOANG00000010091		XP_001506048
Chicken	*Gallus gallus*			XP_424811
Turkey	*Meleagris gallopavo*			XP_019466012
Japanese quail	*Coturnix japonica*			XP_015704470
Swan goose	*Anser cygnoides domesticus*			XP_013052847
Zebra finch	*Taeniopygia guttata*			XP_012433182
Tibetan ground-tit	*Pseudopodoces humilis*			XP_014107713
Hooded crow	*Corvus cornix*			XP_010391160
Peregrine falcon	*Falco peregrinus*			XP_013158040
Brown kiwi	*Apteryx australis mantelli*			XP_013812986
Western clawed frog	*Xenopus tropicalis*			XP_017946448
Common carp	*Cyprinus carpio*			XP_018933702
Channel catfish	*Ictalurus punctatus*		A0A2D0SYD8	XP_017347687
Spotted green pufferfish	*Tetraodon nigroviridis*		Q4T1R6	CAF93166

List of species and NCBI GenBank accession numbers for sequences used to construct the datasets for hypothesis testing.

Supplementary Table 6. The detail information of PD-L2 gene in vertebrate species.

Species	*Scientific Name*	Ensembl	Uniprot	NCBI-Protein ID
Human	*Homo sapiens*	ENSG00000197646	Q9BQ51	NP_079515
Chimpanzee	*Pan troglodytes*	ENSPTRG00000020756		XP_001140776
Bonobo	*Pan paniscus*		A0A2R9AHT0	XP_003823428
Western lowland gorilla	*Gorilla gorilla gorilla*			XP_018889135
Sumatran orangutan	*Pongo abelii*	ENSPPYG00000019236		XP_024107734
Northern white-cheeked gibbon	*Nomascus leucogenys*		G1RCR0	XP_003273875
Rhesus monkey	*Macaca mulatta*		A4GW30	NP_001077068
Crab-eating macaque	*Macaca fascicularis*			XP_005581838
Green monkey	*Chlorocebus sabaeus*	ENSCSAG00000008883		XP_007967513
Golden snub-nosed monkey	*Rhinopithecus roxellana*			XP_010363386
White-tufted-ear marmoset	*Callithrix jacchus*	ENSCJAG00000011422	A0A2R8P957	XP_017828946
Mouse	*Mus musculus*	ENSMUSG00000016498	Q3U304	NP_067371
Rat	*Rattus norvegicus*	ENSRNOG00000016136	D4AAV6	NP_001101052
Chinese hamster	*Cricetulus griseus*			XP_003511103

Mole Rat	*Nannospalax galili*			XP_017658223
American beaver	*Castor canadensis*			XP_020015183
Rabbit	*Oryctolagus cuniculus*			XP_017202816
Pangolin	*Manis javanica*			XR_001852674.1
Dog	*Canis familiaris*	ENSCAFG00000002121	44336	XP_013973349
Giant panda	*Ailuropoda melanoleuca*	ENSAMEG00000009830		XP_011228695
Polar bear	*Ursus maritimus*		A0A384C2M8	XP_008689126
Pacific walrus	*Odobenus rosmarus divergens*		A0A2U3ZNI5	XP_012420555
Domestic cat	*Felis catus*	ENSFCAG00000009048	L7SSK5	NP_001277173
Amur tiger	*Panthera tigris altaica*			XP_007094211
Cheetah	*Acinonyx jubatus*			XP_014919966
Cow	*Bos taurus*		A0A024FBV6	NP_001278965
Wild yak	*Bos mutus*			XP_014332357
Chiru	*Pantholops hodgsonii*			XP_005983898
Goat	*Capra hircus*			XP_013821401
Pig	*Sus scrofa*	ENSSSCG00000026305	Q4QTK0	NP_001020391
Wild Bactrian camel	*Camelus ferus*			XP_014415932
Arabian camel	*Camelus dromedarius*			XP_010991730
Yangtze River dolphin	*Lipotes vexillifer*		A0A340X676	XP_007454585
killer whale	*Orcinus orca*			XP_004279193
Horse	*Equus caballus*	ENSECAG00000020578		XP_005605049
Ass	*Equus asinus*			XP_014716221
Brandt's bat	*Myotis brandtii*			XP_014391175
Black flying fox	*Pteropus alecto*			XP_006918437
African savanna elephant	*Loxodonta africana*			XP_023401133
Florida manatee	*Trichechus manatus latirostris*		A0A2Y9QY77	XP_023584363
Opossum	*Monodelphis domestica*	ENSMODG00000015349	F6WY92	XP_007499609
Tasmanian devil	*Sarcophilus harrisii*	ENSSHAG00000004176	G3VNK0	XP_012399521
Platypus	*Ornithorhynchus anatinus*			XP_007653790
Chicken	*Gallus gallus*			XP_004949124
Turkey	*Meleagris gallopavo*			XP_010723930
Japanese quail	*Coturnix japonica*			XP_015704465
Swan goose	*Anser cygnoides domesticus*			XP_013052854
Zebra finch	*Taeniopygia guttata*			XP_002193013
Tibetan ground-tit	*Pseudopodoces humilis*			XP_014107710
Hooded crow	*Corvus cornix*			XP_010391172
Peregrine falcon	*Falco peregrinus*			XP_013157997
Brown kiwi	*Apteryx australis mantelli*			XP_013812991
Western clawed frog	*Xenopus tropicalis*			XP_017946447
Rock pigeon	*Columba livia*		A0A2I0MG20	XP_005499831
Chinese alligator	*Alligator sinensis*		A0A1U8CUP6	XP_014372758
Western painted turtle	*Chrysemys picta*			XP_005296857

List of species and NCBI GenBank accession numbers for sequences used to construct the datasets for hypothesis testing.

Supplementary Table 7. List of primers used for mRNA expression in qPCR of representative vertebrate species.

Species		5'-3'	TM
Gallus gallus	GgPD1-F	CAGCCACGTTAACTCGTCCT	57.5
	GgPD1-R	GTTCCGGATGATCCCAGCAA	57.8
	GgPDL1-F	CTCATTGTGAGTGCCCTCGT	57.6
	GgPDL1-R	CATGCGCGCCCTTATCTTTC	57.3
	GgPDL2-F	CGCAATGGGAAAGCACTCAC	57.4
	GgPDL2-R	CGCATCTGTGATCTTGACGC	56.9
Eumeces chinensis	EcPD1-F	CTGTTTCTGGTGCTCCCAGT	61.6
	EcPD1-R	TGGTGGCATATTCGGTCTGG	61.7
	EcPDL1-F	TGTATTGGTGTCCTGTTGGCA	56.6
	EcPDL1-R	GGTCATTCACTGGAAACCTGC	56.6
	EcPDL2-F	CGCTACCAGGGAAGAGCAAC	58.9
	EcPDL2-R	TGAGACAGCGGTAAGACCCT	58.1
Acipenser schrenckii	AsPD1-F	CAGAGATGGCCACTGCGTAA	61.7
	AsPD1-R	GTCTGTCTGGGAACGGGATG	61.7
	AsPDL1-F	TAATCCCCGTCGCCCTTTTC	57.8
	AsPDL1-R	TGGTGATGCGCTCTGTTAGG	57.7
	AsPDL2-F	TTGTACCCTTTCACTGCGCT	56.9
	AsPDL2-R	CCATTAAGGGCACCGTCTCA	57.5
Manis javanica	MjPD1-F	GAGGACGAGCCTCTGAAGGA	61.5
	MjPD1-R	CGTGGCATACTCGGTCTGTT	62.5
	MjPDL1-F	GTGAAAGTGGAGGAAGA	47.7
	MjPDL1-R	AGGATGGTCAGGAATTG	47.9
	MjPDL2-F	CCATCGGACGGTCTTTCACTA	60.2
	MjPDL2-R	GCCAGCTCCACACTCTAGCA	59.2
Atelerix albiventris	AaPD1-F	GGCTCTCTTGCTTCTGCCTG	59.2
	AaPD1-R	CGACTGTGAGATGTTGGGGG	58.6
	AaPDL1-F	GTGGTGCCGACTACAAGC	56.8
	AaPDL1-R	TGGGGTAGCCCTCAGCCT	59.1
	AaPDL1-F	GGAGGGCCTGTTGACTTAGG	61.5
	AaPDL1-R	TCTGACTTCCAGGGTCAGGT	61.7
